# Combined expressional analysis, bioinformatics and targeted proteomics identify new potential therapeutic targets in glioblastoma stem cells

**DOI:** 10.18632/oncotarget.4613

**Published:** 2015-07-20

**Authors:** Biljana Stangeland, Awais A. Mughal, Zanina Grieg, Cecilie Jonsgar Sandberg, Mrinal Joel, Ståle Nygård, Torstein Meling, Wayne Murrell, Einar O. Vik Mo, Iver A. Langmoen

**Affiliations:** ^1^ Vilhelm Magnus Laboratory for Neurosurgical Research, Institute for Surgical Research and Department of Neurosurgery, Oslo University Hospital, Oslo, Norway; ^2^ SFI-CAST Biomedical Innovation Center, Oslo University Hospital, Oslo, Norway; ^3^ Bioinformatics Core Facility, Institute for Medical Informatics, Oslo University Hospital and University of Oslo, Oslo, Norway; ^4^ Norwegian Center for Stem Cell Research, Department of Immunology and Transfusion Medicine, Oslo University Hospital, Oslo, Norway; ^5^ Laboratory of Neural Development and Optical Recording (NDEVOR), Department of Physiology, Institute of Basic Medical Sciences, University of Oslo, Oslo, Norway

**Keywords:** glioblastoma, GBM, glioblastoma stem cells, GSCs, therapeutic targeting

## Abstract

Glioblastoma (GBM) is both the most common and the most lethal primary brain tumor. It is thought that GBM stem cells (GSCs) are critically important in resistance to therapy. Therefore, there is a strong rationale to target these cells in order to develop new molecular therapies.

To identify molecular targets in GSCs, we compared gene expression in GSCs to that in neural stem cells (NSCs) from the adult human brain, using microarrays. Bioinformatic filtering identified 20 genes (*PBK/TOPK, CENPA, KIF15, DEPDC1, CDC6, DLG7/DLGAP5/HURP, KIF18A, EZH2, HMMR/RHAMM/CD168, NOL4, MPP6, MDM1, RAPGEF4, RHBDD1, FNDC3B, FILIP1L, MCC, ATXN7L4/ATXN7L1, P2RY5/LPAR6* and *FAM118A*) that were consistently expressed in GSC cultures and consistently not expressed in NSC cultures. The expression of these genes was confirmed in clinical samples (TCGA and REMBRANDT). The first nine genes were highly co-expressed in all GBM subtypes and were part of the same protein-protein interaction network. Furthermore, their combined up-regulation correlated negatively with patient survival in the *mesenchymal* GBM subtype. Using targeted proteomics and the COGNOSCENTE database we linked these genes to GBM signalling pathways.

Nine genes: *PBK, CENPA, KIF15, DEPDC1, CDC6, DLG7, KIF18A, EZH2* and *HMMR* should be further explored as targets for treatment of GBM.

## INTRODUCTION

Glioblastoma (GBM) is the most frequent primary brain tumor. Patient prognosis is poor because tumor cells infiltrating brain tissue surrounding the tumor elude surgery, and adjuvant treatment with irradiation and chemotherapy has only a moderate effect on these remaining cells. As a result, median survival is less than one year [1], although 15 months is reported for selected patients in some clinical trials [2].

Using methods developed to investigate neural stem cells (NSCs) from the adult human brain [3–6], we and others have isolated and propagated stem-like cells from GBMs [7–11]. Dissociated GBM biopsies grown as free-floating tumorspheres in serum-free medium containing mitogens epidermal growth factor (EGF) and basic fibroblast growth factor (FGF) are highly enriched for GBM stem cells (GSCs) [10, 11]. Furthermore, tumor cells derived from these spheres bear genotypic resemblance to the original tumor to a greater extent than serum-cultured cell lines [12]. Upon xenografting, GSCs can restore the phenotype of the original tumor [11] and this ability is sustained even after serial transplantations [8]. GSCs are thought to be responsible for the persistence of GBM growth following therapy [13]. They exhibit efficient protective mechanisms, such as multidrug resistance and DNA repair enzymes that protect them against cytostatic drugs and irradiation [14, 15]. Current therapy therefore mainly targets tumor bulk, thus resulting in a relative enrichment of GSCs [14, 15]. The cancer stem cell hypothesis predicts that these cells must be eradicated in order to obtain a cure [16, 17]. Consequently there is a need for identifying specific molecular targets within GSCs.

The non-cancerous cell type that most closely resembles the GSC is the NSC. NSCs can differentiate into astrocytes, oligodendrocytes and fully functional neurons [3, 4], and do not form tumors following transplantation to the mouse brain [7, 18].

To identify therapeutic targets in GSCs, we combined experimental techniques for analysis of gene and protein expression with public database mining. The increased expression of the selected candidate genes at RNA and protein levels was verified in GSC cultures from independent patient cohorts using qPCR, western blot and immunolabeling. Combining all our results, we found that the increased expression of nine genes (*PBK, CENPA, KIF15, DEPDC1, CDC6, DLG7, KIF18A, EZH2* and *HMMR*) in GSCs and GBM tissues was confirmed with all experimental and bioinformatic methods. They were highly co-expressed in all GBM subtypes and their combined up-regulation correlated with poor patient survival. Thus, there is a strong rationale to explore these nine genes as targets for treatment of GBM.

## RESULTS

### Identification of genes consistently expressed in GSCs and consistently not-expressed in NSCs using microarray

**Figure 1:**
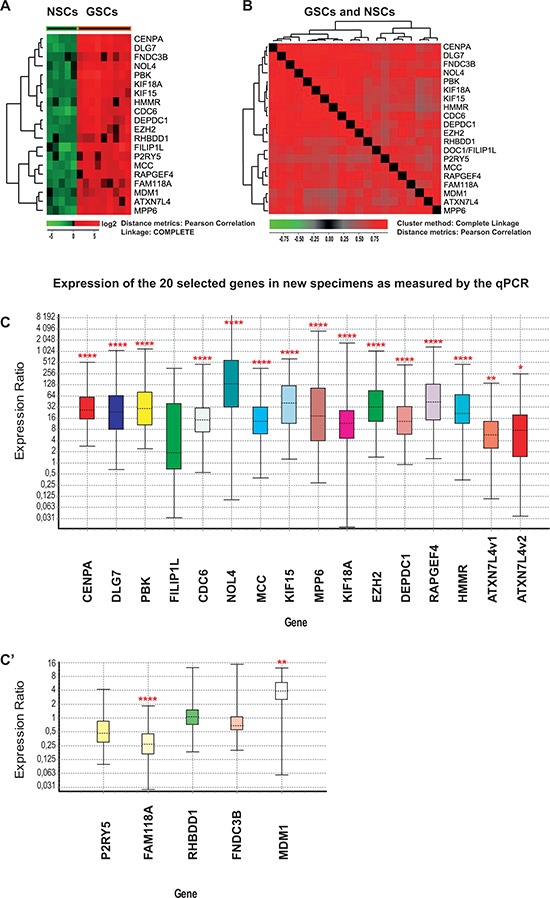
Expression of the 20 selected genes in NSC and GSC cultures measured by microarrays (A-B) and qPCR (C-C’) **A.** Hierarchical clustering of the 20 selected genes in NSC (green) and GSC cultures (red) using Pearson correlation as a distance metric. Gene expression was analyzed in 14 primary cell cultures from newly harvested specimens (nine GSC cultures and five NSC cultures). Red corresponds to higher gene expression levels. **B.** Hierarchical clustering with distance matrix using Pearson correlation as a distance measure was calculated for the same set of data as in A. Red corresponds to higher correlation levels. All fields are red thus indicating that the expression levels of the 20 selected genes are highly correlated in all 14 cultures. **C-C’.** Expression of the 20 selected genes in an independent set of samples measured by qPCR. Four NSC and seven GSC primary cultures were prepared from biopsies of newly harvested tissues. All genes were significantly up-regulated in GSC cultures with the exception of *FILIP1L, P2RY5, RHBDD1* and *FNDC3B*. *FAM118A* was significantly down-regulated. The two isoforms of *ATXN7L4* are indicated as *ATXN7L4*v1 and *ATXN7L4*v2. Expression values in GSCs were calculated using multiple controls (values obtained for all tested NSCs and NFCs) as reference. Fold change values and statistical parameters can be found in Table 1 and Supplementary Table S1. The bottom and top of each box indicate the 25th and 75th percentile (the lower and upper quartiles, respectively), and the band near the middle of the box represents the 50th percentile (the median). The ends of the whiskers represent the minima and maxima of all the data. For data analysis and the preparation of this figure we used the Pfaffl *et al*., 2002 algorithm utilized in REST software [61]. Asterisks correspond to *p* values and indicate level of significance: * = (*p* ≈ 0.01–0.05), ** = (*p* ≈ 0.001–0.01) and **** =(*p* < 0.0001).

In our previous work, we compared GSCs to NSCs from the adult human brain and found a 30-gene signature of highly expressed genes characteristic for GSCs and for several signaling pathways such as the Wnt pathway, that were dysregulated in GSCs [19]. The aim of the current study was to identify genes that can serve as potential therapeutic targets in GSCs. To find genes consistently not expressed in NSCs and consistently expressed in GSCs, we applied a selection rule picking out genes with log2 expression value below zero in all NSC cultures and log2 expression value above zero in all GSC cultures. This rule yielded 20 genes: *PBK/TOPK, CENPA, KIF15, DEPDC1, CDC6, DLG7/DLGAP5/HURP, KIF18A, EZH2, HMMR/RHAMM/CD168, NOL4, MPP6, MDM1, RAPGEF4, RHBDD1, FNDC3B, FILIP1L, MCC, ATXN7L4/ATXN7L1, P2RY5/LPAR6* and *FAM118A* (Figure 1A, Table 1 and Supplementary Figure S1). These genes were all highly co-expressed in all samples as illustrated by hierarchical clustering using Pearson correlation as a distance metric (Figure 1B). These initial findings were then confirmed with a number of experimental techniques and public database mining in order to make a final selection of potentially interesting genes.

**Table 1:** Overview of the expressional analyses and bioinformatics results

	GENE ID	Full name	Microarray		qPCR		Western		Correl. RNA/prot.	Survival	GBM tissues	GBM vs. LGG
			Fold change	Result	Fold change	Result	Fold change	Result	Result	Result	Result	Result
1	CENPA	Centromere protein A.	38.5	UP	39.2	UP	8.0	UP	0.76	Y	Y	Y
2	DLG7	Discs. large homolog 7 (Drosophila)	36.0	UP	24	UP	41	UP	0.7	Y	Y	Y
3	PBK	PDZ binding kinase	40.7	UP	38.1	UP	24.6	UP	0.74	Y	Y	Y
4	FILIP1L	Filamin A interacting protein 1-like	16.1	UP	4.5	NS	31.7	UP	0.73	Y	Y	Y
5	CDC6	CDC6 cell division cycle 6 homolog	26.2	UP	9.2	UP	8.8	UP	0.66	Y	Y	Y
6	NOL4	Nucleolar protein 4	13.5	UP	30.5	UP	7.1	UP	0.7	N	Y	N
7	MCC	Mutated in colorectal cancers	12.4	UP	8.1	UP	0.3	DR	0.42	Y	Y	Y
8	KIF15	Kinesin family member 15	13	UP	62.7	UP	4.7	UP	0.73	Y	Y	Y
9	MPP6	Membrane protein. palmitoylated 6	10.6	UP	9.8	UP	4.6	UP	0.8	Y	Y	N
10	KIF18A	Kinesin family member 18A	12.8	UP	11	UP	22.4	UP	0.89	Y	Y	Y
11	EZH2	Enhancer of zeste homolog 2 (Drosophila)	13.7	UP	35.3	UP	17.1	UP	0.92	Y	Y	Y
12	DEPDC1	DEP domain containing 1	12.2	UP	15.7	UP	49.9	UP	0.52	Y	Y	Y
13	RAPGEF4	Rap guanine nucleotide exchange factor (GEF) 4	9.1	UP	33.4	UP	0.2	DR	0.59	N	Y	N
14	HMMR	Hyaluronan-mediated motility receptor	10.0	UP	25	UP	7.7	UP	0.86	Y	Y	Y
15	ATXN7L4	Ataxin 7-like 4|ataxin 7-like 1	7.4	UP	3.2	UP	0.9	DR	0.4	N	Y	Y
16	P2RY5	Purinergic receptor P2Y. G-protein coupled. 5	5.7	UP	0.5	NS	0.1	DR	0.52	Y	Y	Y
17	FAM118A	Family With Sequence Similarity 118. Member A	3.2	UP	0.3	DR	7.3	UP	0.61	Y	Y	N
18	RHBDD1	Rhomboid domain containing 1	3.3	UP	1.2	NS	2.3	UP	0.05	N	Y	Y
19	FNDC3B	Fibronectin type III domain containing 3B	3.3	UP	0.8	NS	17.5	UP	0.63	Y	Y	Y
20	MDM1	Mdm4. transformed 3T3 cell double minute 1. p53 binding protein (mouse)	3.4	UP	3.4	UP	30.4	UP	0.88	N	Y	Y

Expression of the 20 selected genes in GSC and NSC cultures, measured by microarrays, qPCR and western blot is presented. The results of bioinformatics analysis are presented as: -impact of the gene expression on survival, -expression in GBM tissues and LGG and as correlation between protein and RNA expression (Pearson *r*).

Y = yes; N = no; UP = up-regulated; DR = down-regulated; NS = Not significant. Additional information and statistical parameters can be found in Supplementary Table S1.

### Confirmation of gene expression in an independent set of samples

To confirm the expression of the 20 selected genes, we used real-time quantitative reverse-transcription PCR (qPCR) analysis on independent sets of freshly isolated samples: seven new GSC and four new NSC cultures (Figure 1C–1C’). For each gene, one to seven oligonucleotide primer sets, approximately 1 kb apart from one another, were designed and tested in three GSC and two NSC cultures (Supplementary Figure S2). For the main analysis, only the best performing set was selected. The expression of the selected 20 genes was also tested in a neural fetal cell (NFC) line (ReNcell, Millipore) (Supplementary Figure S3). qPCR analysis was performed on seven new GSC cultures using multiple NSC cultures and a NFC line as reference (Figure 1C–1C’, Table 1 and Supplementary Table S1). It showed that 15 of the 20 selected genes (*PBK, CENPA, KIF15, DEPDC1, CDC6, DLG7, KIF18A, EZH2, HMMR, NOL4, MCC, MPP6, RAPGEF4, ATXN7L4* and *MDM1*) were significantly up-regulated while *FAM118A* and *P2RY5* were down-regulated (Figure 1C–1C’). We did not observe differential regulation of *FILIP1L, RHBDD1* and *FNDC3B* by qPCR. We also calculated the Pearson correlation (PPMCC “*r*”) coefficient between fold change values on microarrays and qPCR. Average correlation for all genes was *r* = 0.51, while the best correlation (*r* = 0.94) was observed for the following genes: *CENPA, DLG7, PBK, MCC, MPPG, KIF18A* and *DEPDC1*.

Differences in culturing conditions are known to influence RNA and protein expression levels, as well as causing differentiation of GSCs [20]. Before analyzing the expression of the 20 selected genes in the new GSC cultures by qPCR, we had to establish a proper set of controls with matching differentiation state, growth parameters and gene expression. The control set consisted of NSCs from different parts of the brain that were grown in three alternative ways, and an NFC line. When cultured as neurospheres, NSCs from the normal human brain typically grow slower than GSCs [7]. As several of the 20 selected genes are involved in cell division and the cell cycle, we questioned whether these genes were up-regulated in GSCs solely because of increased growth rates. We therefore, utilized three alternative protocols to cultivate NSCs: 1. free-floating neurospheres [3, 21], 2. adherent monolayers in a slightly modified neurosphere medium containing 1% serum, bFGF and TGFα (*AD1%* medium) [22], and 3. cells cultured on retronectin-coated wells containing serum-free neurosphere medium [23]. This last protocol has previously only been used for mouse cells.

**Figure 2:**
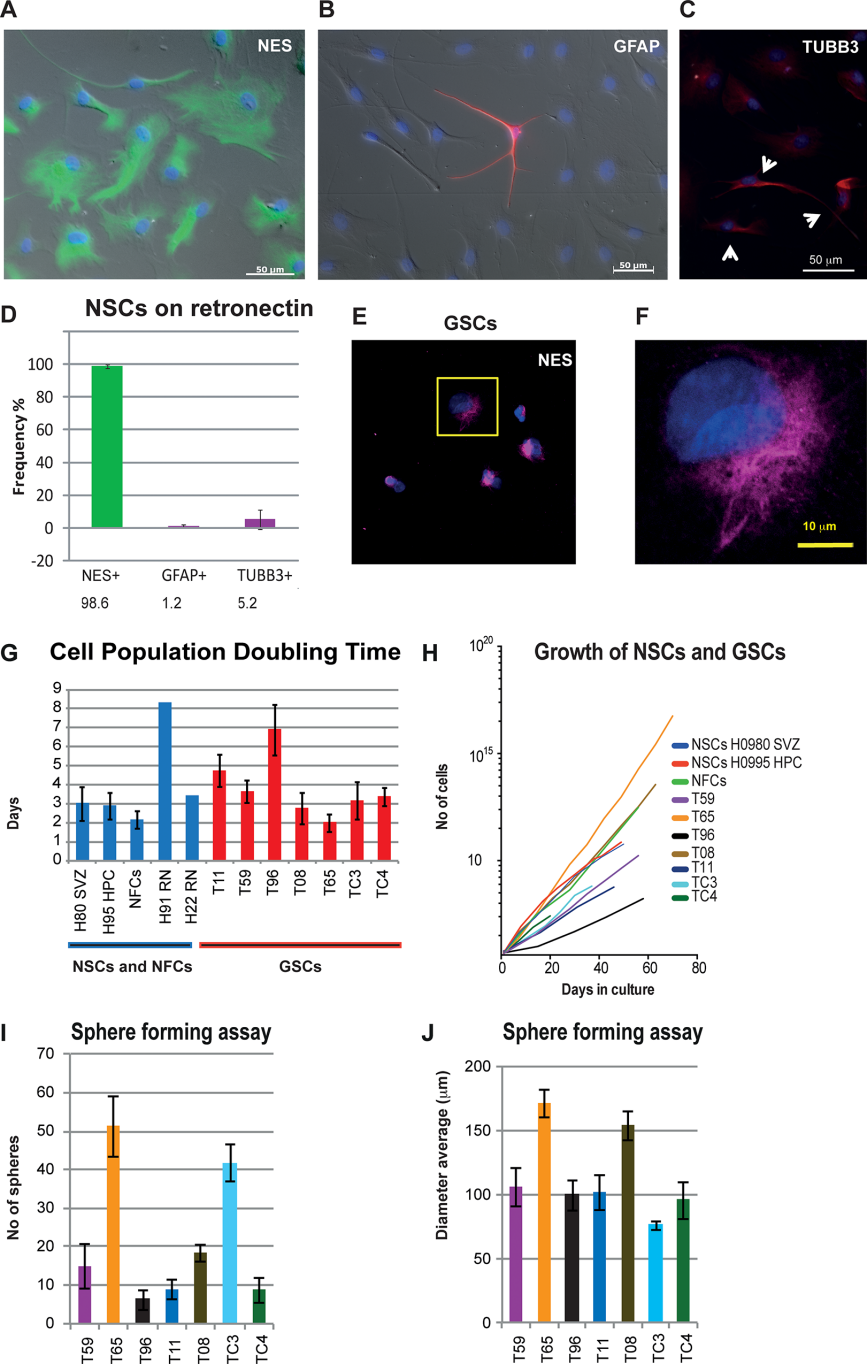
Characterization of state of differentiation and growth parameters in NSCs, NFCs and GSCs **A–D.** NSC cultures incubated on RN remained predominantly undifferentiated. Short incubation (up to a few weeks) on RN resulted in NSC cultures that were 99% nestin positive (NES) (**A**) while only 5.2% and 1.2% of cells were TUBB3 (**C**) and GFAP (**B**) positive, respectively. **A.** Immunolabeling with an anti-nestin antibody (green); Nuclear staining Hoechst 33258 (blue). (**B–C**) Weak TUBB3 and GFAP signals (red) were observed in the majority of cells but only the cells with strong staining were counted (**B** and yellow arrows in **C**). **B.** Very strong signal in a single GFAP positive cell (red). **D.** Frequency calculation for NES, GFAP and TUBB3 positive cells. **E.** Expression of NES in GSC culture T08. **F.** Close up from the marked area in **E. G–J.** Growth parameters calculated for NFC, NSC and GSC cultures. **G.** Doubling time of the cell populations (PDT). PDT values for seven GSC cultures, NFCs and NSCs are shown. NSCs were cultured either in *AD1%* medium (H80 SVZ and H95 HPC) or on RN. **H.** Growth curves of the NFC line and NSC and GSC cultures. Cell cultures were passaged for at least three times. **I.** Sphere forming ability of different GSC cultures varied from less than 10 to more than 60. **J.** Average diameter of spheres for GSC cultures was similar in the majority of cultures. In GSC culture T65, the highest number and size of spheres, and smallest PDT values were observed whilst the GSC culture T96 showed slowest growth (fewer spheres and longest PDT). The error bars represent standard deviations.

We found that adult human NSCs incubated on RN in neurosphere medium behaved quite similarly to the NSCs grown according to the other two protocols (Figure 2A–2D). These cultures expressed high levels of nestin and only a small fraction of the cells expressed the differentiation markers glial fibrillary acidic protein (GFAP) and β3-tubulin (TUBB3) (Figure 2A–2D). All three culturing conditions used for human NSCs thus promoted growth of undifferentiated cells and may serve as appropriate controls for GSCs, in further analyses. Comparisons of *NES, GFAP* and *TUBB3* expressions in GSC, NSC and NFC cultures at RNA and protein levels using qPCR and western blot are also presented (Figure 5 and Supplementary Figures S3–S5).

To further test whether NFCs and NSCs grown in different conditions are suitable as controls, we also determined the growth parameters for all cultures. Both the growth curves and the cell population doubling times (PDT) were analyzed (Figure 2G–2J). The average PDT for two NSC cultures incubated on RN was 5.9 ± 2.4 days. The average PDTs for multiple passages of two NSC cultures grown adherently in *AD1%* medium and NFCs grown as spheres were 2.9 ± 0.7 and 2.1 ± 0.5 days, respectively (Figure 2G–2H). In comparison, the PDT for GSC cultures varied between two to eight days (Figure 2G–2H). The PDT values for all our control cell cultures ranged from two to eight days, thus fulfilling the criteria for adequate growth rate controls.

The GSC cultures were further characterized using a sphere-forming assay where the average number and diameter of spheres were calculated (Figure 2I–2J). While the size of the spheres only varied slightly between the GSC cultures, we detected a positive correlation between number of spheres and reciprocal values of PDTs (*r* = 0.88) (Figure 2G and 2I).

The expression of the selected 20 genes did not differ significantly between NSCs derived from three different anatomical locations or cultured under three different conditions (Supplementary Figures S3, S4 and S5). Even the NSCs, cultured adherently in *AD1%* medium, with PDT comparable to the fastest growing GSC cultures, did not express increased levels of these genes (Figure 2G and Supplementary Figures S5).

Our analysis thus showed that NSCs and NFCs represent suitable controls for our experiments with GSCs, both in terms of growth parameters and differentiation state. Neither the growth rates of the cells nor the immortalization of NFCs significantly influenced the expression levels of the 20 selected genes. This indicates that the increased expression of the tested genes is an inherent feature of GSCs.

### Expression of the 20 selected genes in tissue samples from GBMs and LGGs

**Figure 3:**
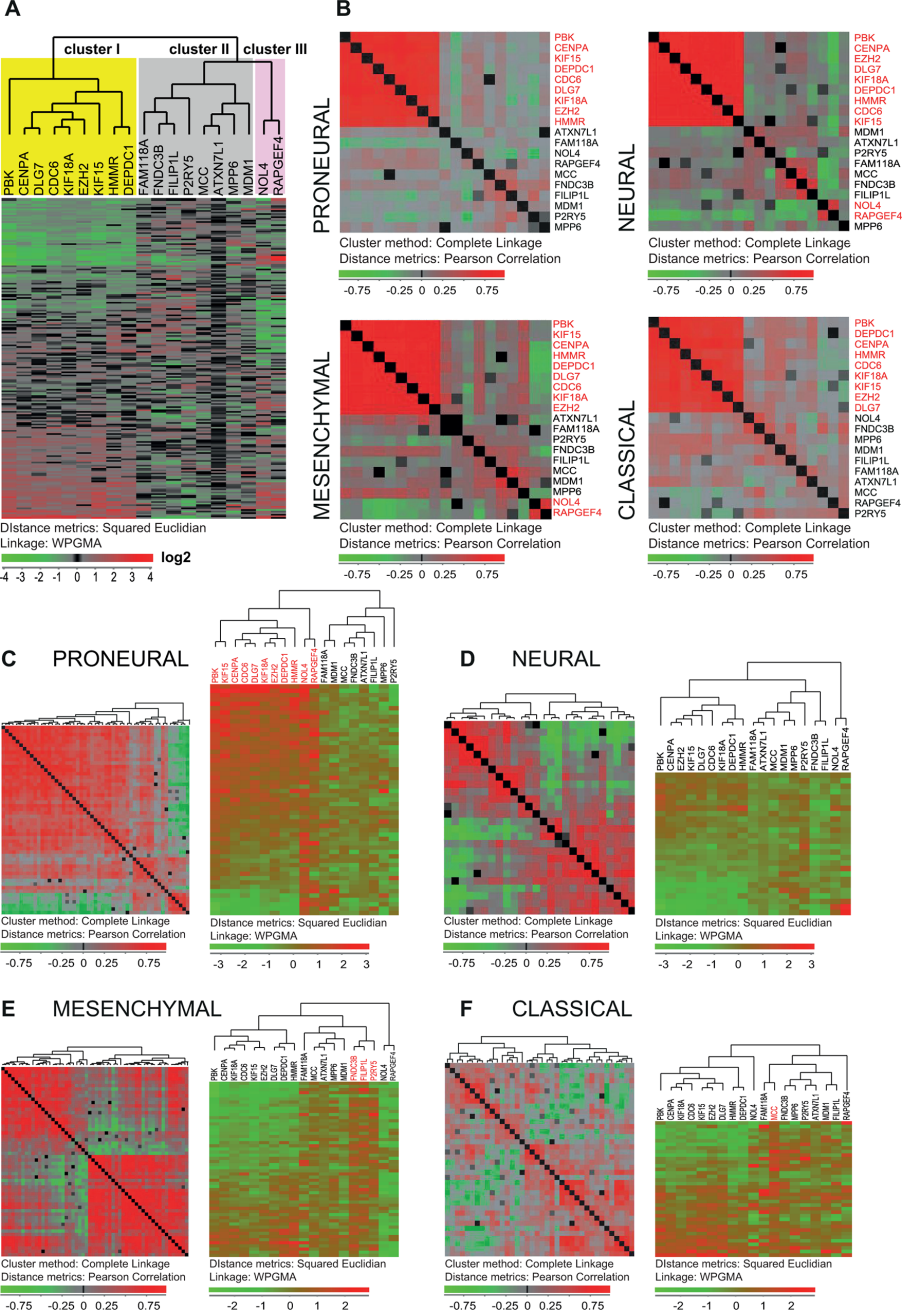
Expression of the 20 selected genes in GBM tissue specimens **A.** Expression of the 19 selected genes (*RHBDD1* was not represented) in the set of 200 GBM tissue samples from the TCGA database [24] is presented as a hierarchical clustering chart. The genes are divided into three groups according to their expression. **B.** Expression of the 19 genes calculated for each of the GBM subtypes (*proneural, mesenchymal, neural* and *classical*) presented as a hierarchical clustering with distance matrix (Pearson) chart. Each square represents correlation between two genes. Red corresponds to higher correlation levels. This analysis showed that particularly the nine following genes: *CENPA, DLG7, PBK, CDC6, KIF15, KIF18A, EZH2, DEPDC1* and *HMMR* (red text) were highly co-expressed in all four subtypes. **C–F.** Both the hierarchical clustering (Squared Euclidian) and the hierarchical clustering with distance matrix (Pearson) are shown for the 19 genes in each of the GBM subtypes. For panels on the left each square represents the degree of linear dependence (Pearson correlation) between two samples. For the majority of *proneural* tumors (**C**, panel on the left) the degrees of linear dependences between the samples were high thus indicating that the expression of the whole 19-gene set was uniform in the majority of the tissue samples of this GBM subtype. The panel on the right (**C**) shows that especially the nine genes from cluster I (*CENPA, DLG7, PBK, CDC6, KIF15, KIF18A, EZH2, DEPDC1, HMMR*) and the two genes from cluster III, (*NOL4* and *RAPGEF4*) were highly expressed in the majority of *proneural* samples. The expression of these genes was weaker in the *mesenchymal, neural* and *classical* GBM subtypes (**D–F**, right). (**E**) *NOL4* and *RAPGEF4* were lowly expressed while *FND3C, P2RY5* and *FILIP1L* were highly expressed in the *mesenchymal* subtype. *MCC* and *NOL4* were previously characterized as the genes that specify the *classical* (**F**) and *proneural* (**C**) subtypes respectively [24].

We also analyzed the mRNA expression of the 20 selected genes in tissues derived from GBMs and LGGs using the two largest publicly available databases, REMBRANDT (https://caintegrator.nci.nih.gov/rembrandt/) and TCGA (http://cancergenome.nih.gov/). Analysis of the data from these databases confirmed the expression of all 20 genes in GBM tissues (Figure 3, Supplementary Table S2).

Integrated genomic analysis has previously identified four molecular subtypes of GBM: *proneural, neural, mesenchymal* and *classical* [24]. To determine the expression of the selected 20 genes within the GBM subtypes, we used a formerly described gene expression data set of 200 GBM samples from TCGA [24]. By applying hierarchical clustering to the entire set of GBM samples, 19 of the genes (RHBDD1 was not represented) could be grouped into three clusters according to their mRNA expression levels (Figure 3A). The first cluster included nine genes (*CENPA, DLG7, PBK, CDC6, KIF15, KIF18A, EZH2, DEPDC1* and *HMMR*), the second eight genes (*FILIP1L, MCC, MPP6, ATXN7L4, P2RY5, FAM118A, FNDC3B* and *MADM1*), and the third two genes (*NOL4, RAPGEF4*) (Figure 3A). While the expression levels of the whole 20-gene set seemed to be highly correlated in the GSC and NSC cultures (Figure 1B), only the nine genes in cluster I seemed to be consistently co-expressed in all GBM subtypes in tissue samples from tumor bulk (Figure 3B). Our analysis thus identified a co-expression module (cluster I) characteristic for both GSCs and GBM (Figures 1A and 3B). When it comes to the level of expression this nine-gene co-expression module was particularly highly expressed in *proneural* tumors and relatively weakly expressed in *neural* tumors (Figure 3C–3D). *NOL4* and *RAPGEF4* (cluster III) were highly expressed in *proneural* and weakly expressed in *mesenchymal* tumors (Figure 3C–3D). *FNDC3B* and *FILIP1L* were weakly expressed in the *neural* subtype (Figure 3D). *FNDC3B, FILIP1L* and *P2RY5* (cluster II) were consistently up-regulated in *mesenchymal* tumors (Figure 3E). Only *NOL4* and *MCC* have previously been identified as typical *proneural* and *classical* gene reporters, respectively [24]. This is in accordance with our results (Figure 3C and 3F).

We further compared GBMs to LGGs using the gene expression dataset from REMBRANDT. At a FDR of 10%, 16 of our 20 genes were represented with 34 probes. Out of these 16 genes, 11 were up-regulated in GBMs compared to LGGs (*CENPA, DLG7, PBK, CDC6, KIF15, KIF18A, EZH2, DEPDC1, HMMR, FILIP1L* and *FNDC3B*) (Supplementary Table S2).

### Correlation between gene expression and patient survival

**Figure 4:**
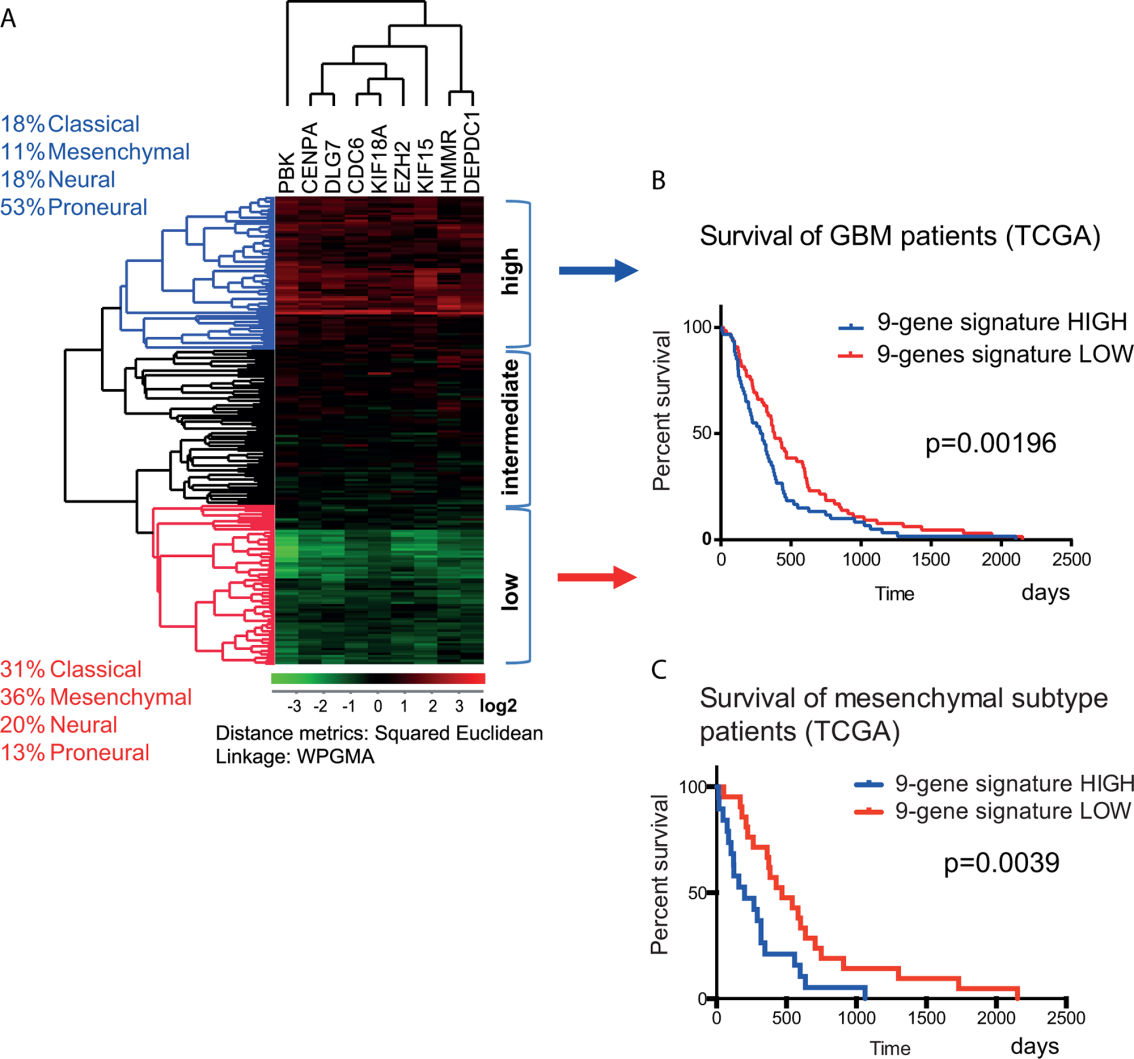
Correlation between gene expression and the survival of GBM patients **A.** The correlation between gene expression and survival was calculated using the set of 200 GBM samples from the TCGA database described in Verhaak *et al*., 2010 [24]. This material contained 54 *classical*, 58 *mesenchymal*, 57 *proneural* and 33 *neural* GBM tissue samples. Using hierarchical clustering, the patients were sorted according to the expression of the nine genes in gene cluster I (from Figure 3A). **B.** The survival times of GBM patients with the highest and lowest expression of the nine genes were compared. The subclassification of these patient groups is described in the text on the left (high = blue and low = red). This analysis showed that the concurrent high expression of the nine genes had a negative effect on patient survival (median survival reduced from 383 to 290 days). The calculated *p* value was *p* = 0.00196 (Gehan-Breslow-Wilcoxon test). **C.** The survival times of *mesenchymal* subtype of GBM patients with the highest and lowest expression of the nine genes were compared (median survival reduced from 468 to 199 days). The calculated *p* value was *p* = 0.0039 (Gehan-Breslow-Wilcoxon test).

To explore the correlation between clinical outcome and the expression levels of the 20 selected genes, we performed analyses based on the REMBRANDT and TCGA datasets. The gene expression analysis of the 200 GBM samples [24] from TCGA showed that the combined expression of the nine genes in cluster I had a significant effect on survival (Figure 4B–4C). Based on the mRNA expression levels of these nine genes, the GBMs from TCGA samples were divided into three groups: low (green), intermediate (black) and high expression (red; Figure 4A). Kaplan-Meier survival analysis showed that concurrent up-regulation of these nine genes correlated with a moderate but significant decrement in survival of GBM patients (Figure 4B). Further analysis of the survival in the GBM subtypes from TCGA revealed that the concurrent up-regulation of the nine genes correlated negatively with patient survival in the *mesenchymal* (Figure 4C) but not in the other GBM subtypes (not shown). The Kaplan-Meier graph showed reduction in median survival from 468 to 199 days in *mesenchymal* GBM patients upon increased expression of the nine genes (Figure 4C).

The expression of the 20 selected genes and patient survival were also analyzed in the REMBRANDT dataset for groups including a) all gliomas irrespective of histological grade, and b) LGGs (predominantly grade II). The expression values for 51 probes representing 18 of our genes were significant and could predict survival (not shown). When considering glioma patients as one group irrespective of histological grade, the increased expression of 15 genes (*CENPA, DLG7, PBK, FILIP1L, CDC6, MCC, KIF15, MPP6, KIF18A, EZH2, DEPDC1, HMMR, P2RY5, FAM118A*, and *FNDC3B*) correlated with the least favorable survival (Supplementary Figure S6A). In addition, *FNDC3B* was found to be a significant predictor of survival within the LGG group (Supplementary Figure S6B). The small size of the GBM sample set in the REMBRANDT database limited Kaplan-Meier analysis to only a few genes (Supplementary Figure S6, table).

### Confirmation of expression at protein level, targeted proteomics and regulatory networks

**Figure 5:**
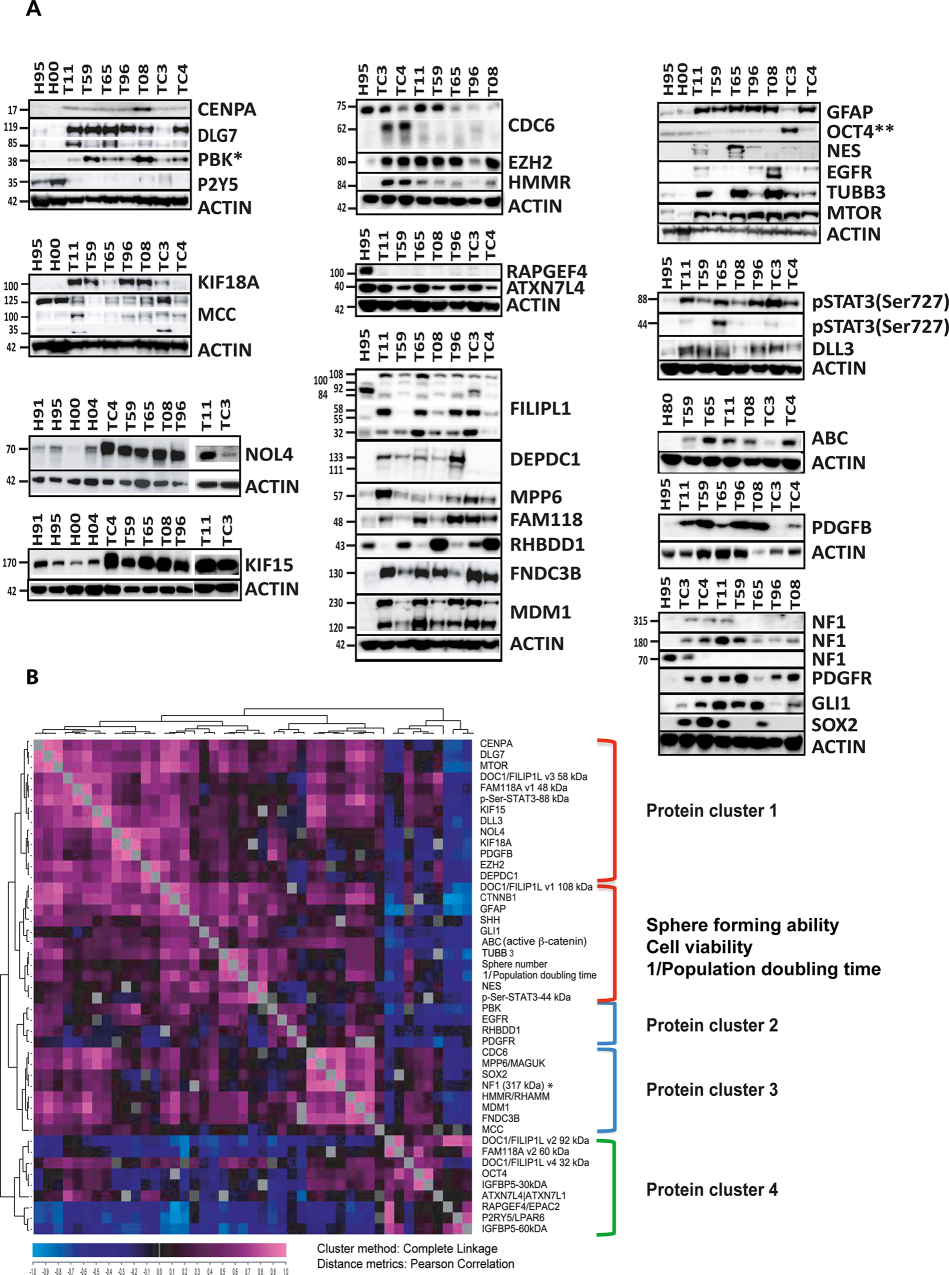
Expression of the proteins encoded by the 20 selected genes and targeted proteomics **A.** Western blot showed that 15 genes were significantly up-regulated at the protein level in all tested GSC cultures, including CENPA, DLG7, PBK, FILIPL1, DEPDC1, NOL4, CDC6, KIF15, MPP6, KIF18A, EZH2, HMMR, FAM118A, FNDC3B and MDM1. RHBDD1 was up-regulated at the protein level in some of the GSC cultures while MCC and RAPGEF4 were clearly down-regulated at the protein level even though their RNA expression levels were significantly higher in GSCs. This analysis was performed in two to four NSC (not all shown) and seven GSC cultures. The observed sizes in kDa are indicated. The expected protein sizes, quantification of the observed bands and additional information can be found in Table 1 and Supplementary Figure S7. *This western blot was included in another manuscript [56]. **This western blot was previously published in [22]. **B.** Results of targeted proteomics. Quantified western data were used for hierarchical clustering with a distance matrix in order to determine the level of co-expression. Each square in the chart represents the Pearson's correlation between the expression levels of two proteins (pink representing the highest and dark blue the lowest correlation). Reporters of the known signaling pathways and proteins relevant for sub-classification of GBM at the protein level [26] were also included (western images of the reporters are shown in A, panel to the right). In addition to the western data, the results of the functional assays were quantified and added to the protein dataset. The sphere forming assay and PDT values are presented as normalized values (0–1, 1 being the highest sphere forming ability, 0 being the number of spheres in NSCs) and reciprocal values (1/n), respectively. The nine proteins (corresponding to the nine genes in gene cluster I) are indicated in yellow.

To further explore the potential for therapeutic targeting, we analyzed the expression of the proteins encoded by the 20 selected genes using western blot, immunolabeling, targeted proteomics and bioinformatics. Western blot showed that 15 of the proteins encoded by the 20 selected genes were up-regulated in all tested GSC cultures: CENPA, DLG7, PBK, FILIPL1, DEPDC1, NOL4, CDC6, KIF15, MPP6, KIF18A, EZH2, HMMR, FAM118A, FNDC3B and MDM1 (Figure 5A, Supplementary Figure S7). Two of the five remaining proteins were not differentially regulated at the protein level (RHBDD1 and ATXN7L4), while three were down-regulated (P2Y5, MCC and RAPGEF4) in GSC cultures (Figure 5A, Supplementary Figure S7). It has been shown that the protein amount can not always be deduced by measuring mRNA levels [25]. To calculate correlation between the mRNA and the corresponding protein levels, we quantified western data and translated them into relative protein expression (RPE) values (Figure 5A, Table 1). These were used to calculate Pearson correlation between RNA and protein expression for each gene (for details see Material and Methods section). For 17 genes (*PBK, CENPA, KIF15, DEPDC1, CDC6, DLG7, KIF18A, EZH2, HMMR, NOL4, MPP6, MDM1, FNDC3B, FILIP1L, ATXN7L4, P2RY5* and *FAM118A*), we found good correlation (*r* = 0.64 + 021) between the RNA and the corresponding protein levels (Table 1). For three genes (*RAPGEF4, RHBDD1* and *MCC*), we found negative RNA-protein correlation (*r* = −0.35 ± 0.27). The overall correlation for the entire set of 20 genes was *r* = 0.55 ± 0.41. The nine genes from gene cluster I exhibited very high RNA-protein correlation (*r* = 0.75 ± 0.12).

To evaluate the potential functional association between the proteins encoded by the 20 selected genes and important signaling pathways in GSCs we used a targeted proteomics approach (Figure 5A–5B). Through hierarchical clustering with distance matrix (Pearson), we analyzed the expression of a) proteins encoded by the 20 selected genes, b) several principal regulators of stemness and tumorigenicity in GSCs, c) reporters of the signaling pathways relevant for GBM such as: active CTNNB1 (ABC) (Wnt pathway), SHH and GLI1 (Hedgehog pathway), MTOR (MTOR pathway), DLL3 (Notch pathway) and STAT3 (Jak/stat pathway), d) proteins relevant for sub-classification of GBMs/GSCs at the protein level [26] and e) the reporters of growth factor signaling pathways (IGFBP5, PDGFB, EGFR and NF1). In addition to the RPE values obtained using seven GSC cultures and two NSC cultures, we also included reciprocal values of PDT and the results from functional assays (cell viability/XTT and sphere forming assay) (Figure 5B). Hierarchical clustering with distance matrix separated the tested proteins into two classes: proteins up-regulated in GSCs (clusters 1, 2 and 3) and those down-regulated in GSCs (cluster 4) (Figure 5B). Gene products of the nine genes in gene cluster I were all up-regulated and co-expressed at the protein level. From these, six proteins (CENPA, DLG7, KIF15, KIF18A, EZH2 and DEPDC1) were highly co-expressed and were arranged within protein cluster 1 together with signaling pathway reporters such as MTOR, pSTAT3 (Ser^727^), DLL3 and PDGFB (Figure 5B). PBK was co-expressed with EGFR and PDGFR (protein cluster 2) while the expression levels of CDC6 and HMMR correlated with one another and with the expression of the stemness marker SOX2 (protein cluster 3). In addition, the expression levels of reporters of the Wnt and Hedgehog signaling pathways correlated positively with levels of nestin, sphere forming ability and PDT (protein cluster 2).

**Figure 6:**
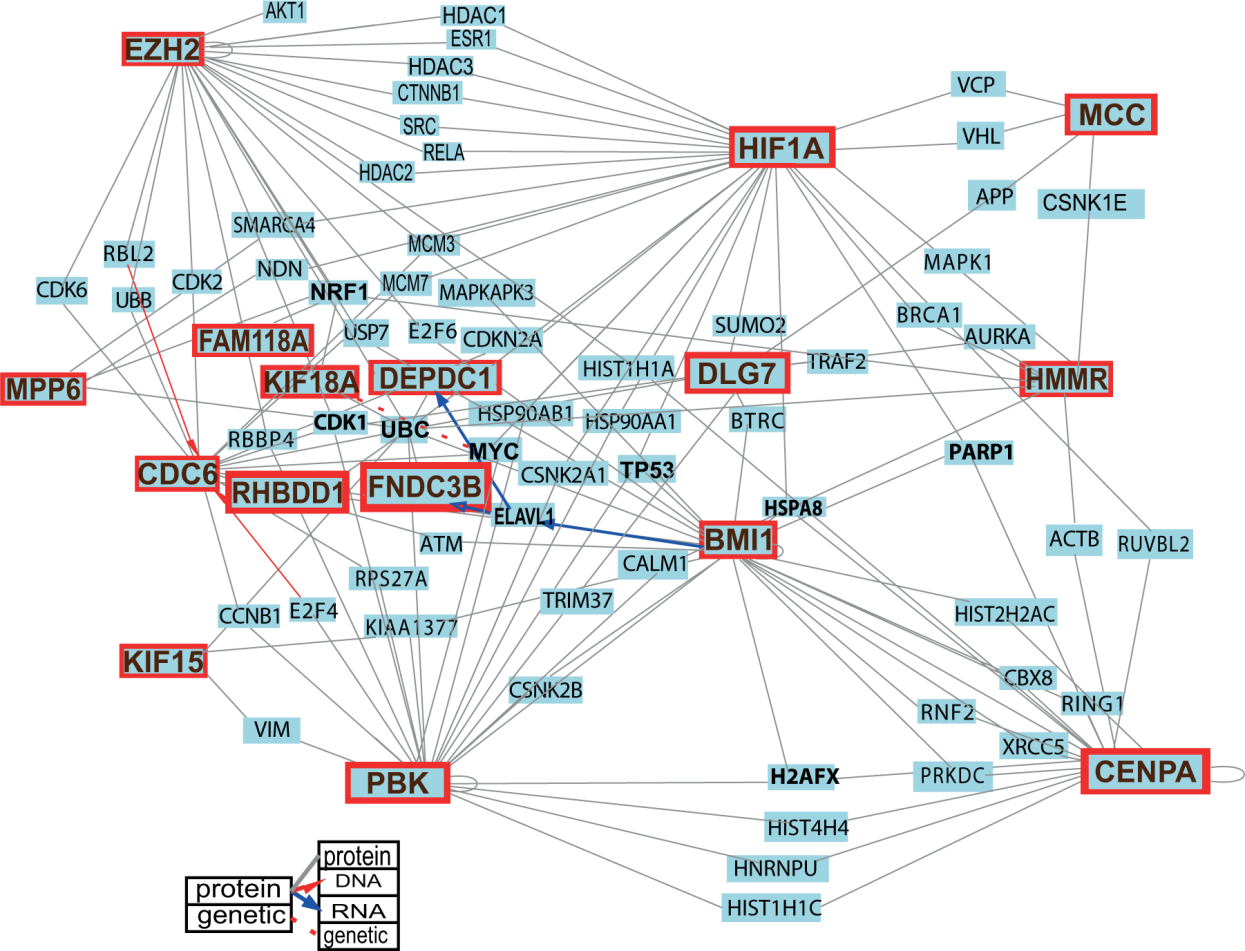
Protein-protein interactions among the proteins encoded by the 20 selected genes and the principal regulators of stemness, growth and tumorigenicity in GSCs By querying the COGNESCENTE database we obtained information on protein-protein interactions documented in the literature. Fourteen of the proteins encoded by the selected 20 genes were interconnected and built a network. Several of these (PBK, CENPA, CDC6, EZH2, MPP6 and MCC) were highly interconnected and could be called network “hubs”. For clarity of the image the list of queried genes was limited to the 20 selected genes, and BMI1 and HIF1A. For a detailed image with all interactions see Supplementary Figure S8.

To investigate whether the results of the targeted proteomics can be confirmed by other means, we searched the COGNOSCENTE database (http://vanburenlab.medicine.tamhsc.edu/cognoscente.shtml) that contains information about biomolecular interactions based on experimental evidence. The interactions between molecules represented in this database included protein-protein, protein-DNA, protein-RNA and genetic interactions. In this analysis, we included proteins used in targeted proteomics (Figure 6, Table 2, Supplementary Figure S8). Analysis of protein cluster 1 (Figure 5B) revealed that MTOR might be functionally related to CENPA and EZH2 with whom it shared three and four common interactants respectively. DLL3 was interconnected with EZH2 and KIF18A via NRF1. STAT3 was interconnected with EZH2 via 13 common interactants, with CENPA via SRRT, with DLG7 via SUMO2 and with DEPDC1 via transcription factor ELAVL1. CENPA and EZH2 were connected via HIST1H1A while DLG7 and EZH2 shared one common interactant CDK1. Furthermore MTOR, STAT3, EZH2, DEPDC1, KIF15, KIF18A and DLG7 were all interconnected via ubiquitin C (UBC). Analysis of protein cluster 2 (Figure 5B) showed that EGFR was interconnected with PBK via 11 common interactants (Table 2) and with RHBDD1 via FBXO25 and UBC. In protein cluster III SOX2 shared common interactants with CDC6, HMMR and MPP6. NF1 was connected with MPP6 via SMARCA4 while NRF1 bound NF1, MPP6 and HMMR. Furthermore NF1, FNDC3B, HMMR, MPP6 and CDC6 were all interconnected via UBC.

**Table 2:** Highlighted protein-protein interactions extracted from the COGNESCENTE database

INTERACTIONS WITHIN CLUSTERS			
	interactants		interaction	
		direct	indirect via
**cluster 1**	MTOR-CENPA	no	RUVBL1, RUVBL2, DDB1
	MTOR-EZH2	no	SIRT1, C7ORF25, PML, AKT1
	DLL3-EZH2	no	NRF1
	DLL3-KIF18A	no	NRF1
	STAT3-EZH2	no	RPS6K5, ESR1, HDAC1, HDAC2, HDAC3, PML, RELA, KDM1A, MYOD1, SRC, MAP3K7, DNMT1, ASXL1
	STAT3-CENPA	no	SRRT
	STAT3-DLG7/DLGAP	no	SUMO2
	STAT3-DEPDC1	no	ELAVL1
	CENPA-EZH2	no	HIST1H1A
	DLG7-EZH2	no	CDK1
	UBC-(MTOR, STAT3, EZH2, DEPDC1, KIF15, KIF18A and DLG7)	yes	
**cluster 2**	EGFR-PBK	no	H2AFX, CALM1, HSP90AB1, HSPA5, HSPA8, HSPA1A, PRDX1, TUBB, HBA1, JUP and CDC37
	EGFR-RHBDD1	no	FBXO25, UBC
**cluster 3**	SOX2-CDC6	no	FZR1, BMI1 and CDK1
	SOX2-HMMR	no	TUBB2A
	SOX2-MPP6	no	SKIV2L2
	NF1-MPP6	no	SMARCA4
	NRF1-(HMMR, NF1, MPP6)	yes	
	UBC-(NF1, FNDC3B, HMMR, MPP6, CDC6)	yes	
**INTERACTIONS ACROSS CLUSTERS**			
**clusters 1, 2 and 3**	EZH2-CDC6	no	CDK2, CDK6, RBL2, UBB
	MCC-DLG7	no	APP
	MCC-HMMR	no	CSNK1E
	HMMR-CENPA	no	ACTB
	PBK-CENPA	no	HNRNPU, H2AFX, HIST4H4, HIST1H1C, HSPA8
	PBK-KIF15	no	KIAA1377, VIM
	PBK-CDC6	no	CCNB1, RPS27A, E2F4 (DNA-protein binding), MYC
	PBK-HMMR	no	CALM1
	PBK-DLG7	no	TRIM37
	PBK-EZH2	no	RBBP4, CDK1
**OTHER INTERACTIONS**			
	HIF1A-EZH2	no	HDAC1, HDAC2, HDAC3, RELA, ESR1, CTNNB1, SRC
	EZH2-STAT3	no	HDAC1, HDAC2, HDAC3, RELA, ESR1,
	HIF1A-CDC6	no	MCM3, MCM7, CDKN2A
	HIF1A-MPP6	no	SMARCA4, NDN
	HIF1A-HMMR	no	BRCA1. MAPK1
	HIF1A-MCC	no	VCP, VHL
	HIF1A-CENPA	no	RUVBL2, PARP1, HSPA8
	HIF1A-PBK	no	CSNK2A1 and HSPA8
	HIF1A-DLG7	no	SUMO2
	BMI1-CENPA	yes	XRCC5, PARP1, CBX8, PRKDC, RING1, RNF2, H2AFX
	BMI1-CDC6	yes	ATM
	BMI1-PBK	no	H2AFX, KIAA137, TP53, CSNK2B
	BMI1-EZH2	no	E2F6, MAPKAPK3 and USP7
	BMI1-PBK	no	H2AFX, KIAA137, TP53, CSNK2B
	BMI1-KIF15	no	KIAA137
	BMI1-DLG7	no	BTRC
	BMI1-MPP6	no	UBC
	SALL2- CENPA	no	DDB1
	SALL2- PBK	no	RBBP7
	SALL2- SOX2	no	RBBP7
	POU3F2-OLIG2	no	SOX10
	POU3F2-OLIG2-HIF1A	no	EP300
	CDK1-(PBK, CDC6, DLG7, EZH2)	yes	

To investigate if the proteins encoded by the 20 selected genes interact with each other across the protein clusters and whether they build a network, we searched the COGNOSCENTE database again. This analysis revealed that proteins encoded by the 14 candidate genes (*PBK, CENPA, KIF15, DEPDC1, CDC6, DLG7, KIF18A, EZH2, HMMR, MPP6, RHBDD1, FNDC3B, MCC*, and *FAM118A*) were functionally interconnected and built a protein interaction network (Figure 6, Table 2, Supplementary Figure S8). Among these, we detected six genes (*PBK, CENPA, CDC6, EZH2, MPP6* and *MCC*), whose gene products interacted with a high number of proteins and qualify to be called high degree nodes or “hubs”. KIF15, HMMR and DLG7 were also highly interconnected within this network. EZH2 and CDC6 were connected via CDK2, CDK6, RBL2, and UBB. MCC interacted with DLG7 via APP and with HMMR via CSNK1E. HMMR was further connected with CENPA via ACTB. PBK was interconnected with CENPA, KIF15, CDC6, HMMR, DLG7 and EZH2 (Table 2).

We also analyzed known interactions between the proteins encoded by the 20 selected genes and two known key players that regulate growth and development of GSCs, namely BMI1 and HIF1A (Figure 6 and Supplementary Figure S8). HIF1A interacted with EZH2 via seven interactants. Five of the interactants were also shared between EZH2 and STAT3. HIF1A further interacted with CDC6, MPP6, HMMR, MCC, CENPA, PBK and DLG7. BMI1 both bound CENPA and interacted with it via seven interactants. BMI1 both bound CDC6 and interacted with it via ATM. BMI1 further interacted with PBK, EZH2, KIF15, DLG7 and MPP6. BMI directly regulated expression of ELAVL1 that further directly regulated expression of DEPDC1 and FNDC3B.

We also looked into interactions between the proteins encoded by the 20 selected genes and products of SOX2, OLIG2, POU3F2 and SALL2 that were recently identified as a core set of transcriptional factors essential for neurodevelopment and GBM propagation [27]. All four proteins were part of the same network (Table 2, Supplementary Figure S9).

One of the proteins central to the network was ubiquitin C (UBC) that bound 11 proteins encoded by the following genes: *PBK, KIF15, DEPDC1, CDC6, DLG7, KIF18A, EZH2, HMMR, MPP6, RHBDD1* and *FNDC3B* (Figure 6, Supplementary Figures S8–S9) as well as with products of *BMI1, MTOR, STAT3, HIF1A, EGFR, NF1, POU3F2* and *SALL2*. Another interconnecting protein was encoded by nuclear respiratory factor 1 (*NRF1)* and bound 5 proteins (EZH2, HMMR, KIF18A, MPP6 and FAM118A) as well as DLL3 and NF1. Cyclin-dependent kinase 1 (CDK1) bound four proteins (PBK, CDC6, DLG7 and EZH2), while beta actin (ACTB) and retinoblastoma binding protein 4 (RBBP4) bound three proteins each. ACTB bound PBK, CENPA and HMMR while RBBP4 bound CDC6, EZH2 and PBK. Moreover, V-myc avian myelocytomatosis viral oncogene homolog (MYC) bound CDC6 and HIF1A and was functionally related to KIF18A and PBK inferred from genetic evidence (Figure 6). RAPGEF4 and NOL4 were not part of this network but were connected to MTOR via RAP1A and to SOX2 via CTBP2 respectively (Supplementary Figure S9). MDM1 and ATXN7L1 bound only with one known protein each (Figure 6).

**Figure 7:**
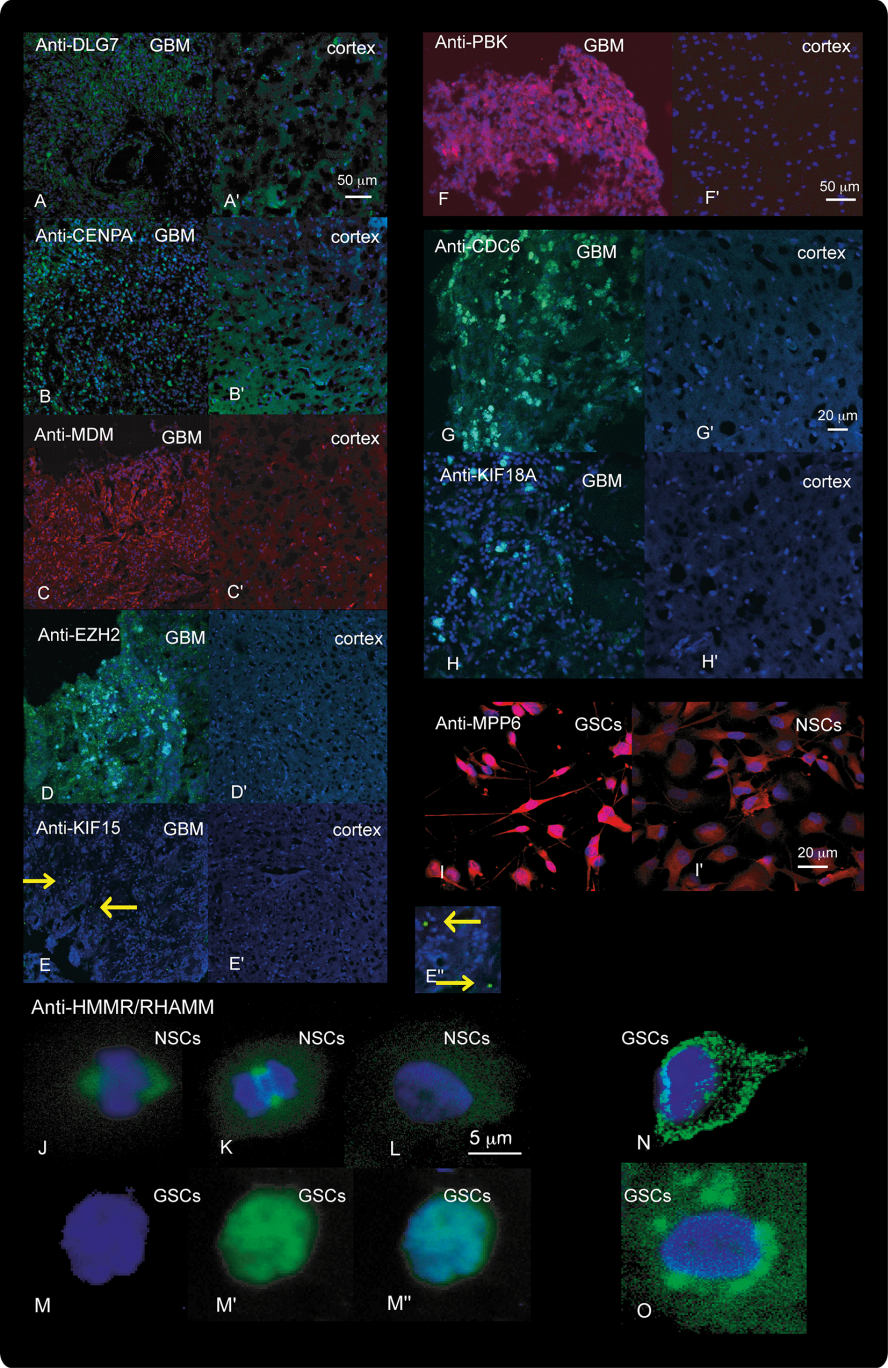
Results of Immunolabeling Immunolabeling with antibodies against DLG7, CENPA, MDM, EZH2, KIF15, PBK, CDC6 and KIF18A in the cerebral cortex (**A’–H’**) and in GBM tissues **A–H.** and against MPP6 and HMMR in NSCs (**I’, J–L.** and GSCs **I, M–O.** is shown. Tissues immunolabeled with anti-DLG7 (**A-A’**), anti-CENPA (**B-B’**), anti-EZH (**D-D’**), anti-KIF15 (**E-E”**), anti-CDC6 (**G-G’**), anti-KIF18A (**H-H’**), and HMMR (**J–O**) were visualized with green fluorescence. Tissues and cells immunolabeled with Anti-MDM1 (**C-C’**), anti-PBK (**F-F’**) and anti-MPP6 (**I-I’**) were visualized with red fluorescence. DAPI staining of the nuclei is visualized as blue fluorescence. **E”**, Enlargement of a section from **e** showing the KIF15 signal (arrowheads) in GSCs. In NSCs the HMMR protein was located in centromeres during mitosis (**J** and **K**) and diffusely spread through the cytoplasm during interphase (**L**). In GBM HMMR was both up-regulated and showed aberrant distribution in the cells (**M–O**). In these cells, HMMR was detected in the cytoplasm (**N**), around the nucleus (**O**) and in the nucleus, where its expression overlapped with DAPI (**M-M”**). **M**, nuclear staining (blue). **M‘** Anti-HMMR staining (green). Overlap between the two (**J, M”, N** and **O**).

Increased protein levels for a set of the selected genes were also confirmed in GSCs, NSCs and GBM tissues using immunolabeling (Figure 7). Interestingly, there were significant differences in the subcellular localization of HMMR in GSCs and NSCs (Figure 7J–7O).

### Global analysis

**Figure 8:**
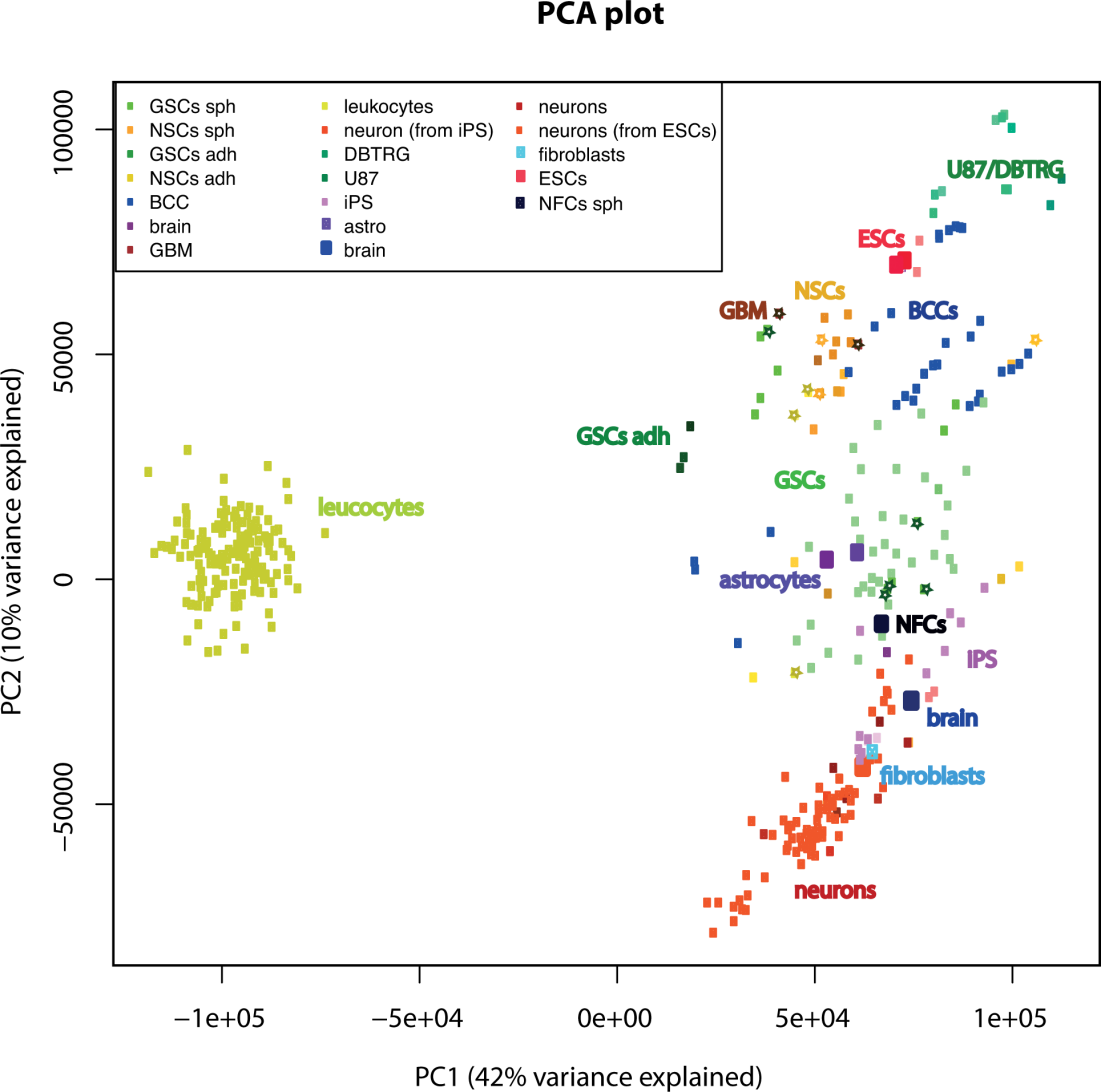
Global analysis comparing GSC and NSC cultures used in this work, to various cell types and tissues. For visualization of global analysis we used principal component analysis (PCA) of gene expression Cultures used in this work are indicated by stars. Abbreviations: DBTRG and U87 are GBM cell lines; BCC-breast cancer cells (both cell lines and cancer stem cells), ESCs-embryonic stem cells; iPS cells-induced pluripotent stem cells; adh-adherent cells.

We have performed a global analysis comparing GSC and NSC cultures and GBM tissues used in this work to iPS cells, neurons, iPS-derived neurons, astrocytes, fibroblasts, NSCs, NFCs, breast cancer cells (BCC), leucocytes, brain tissue, GBM, ESCs and many additional sets of GSCs from the GEO database. For visualization we used principal component analysis (PCA). This analysis showed that our sets of NSCs and GSCs clustered together with other NSCs and GSCs from the GEO database (Figure 8). The first principal component (PC) separated leucocytes from all other cell types. The second PC separated GSCs from differentiated neurons. GSCs were only partially separated from NSCs and BCC.

The GEO data sets were also used to compare expression of the nine potential target genes in GSCs, astrocytes, DA-neurons, iPS-derived neurons, iPS cells and ESCs (Supplementary Figure S11). Eight genes (all except *EZH2*) showed reduced expression in astrocytes when compared to GSCs. Although the trend was similar for all eight genes, statistical significance was not reached in all tests (probably due to the small sample sizes). Eight genes (all except *EZH2*) showed reduced expression in neurons when compared to GSCs. Also here the trend was similar for all eight genes although statistical significance was not reached in all tests. The expression of all genes, except *CDC6*, was relatively high in ESCs. The expression of *DEPDC1* and *HMMR* was significantly higher in ESCs than in GSCs. The expression of *HMMR* and *DLG7* was significantly higher in iPS cells than in GSCs.

## DISCUSSION

It has been shown that GSCs are involved in GBM development and resistance to therapy [13–15]. Hence, there is a strong rationale to target these cells as a therapeutic strategy. By comparing the gene expression levels in NSCs and GSCs, we previously identified a 30-gene signature and pathways that are differentially regulated between these two cell types [19]. In the work presented here, we focused on identifying genes suitable for therapeutic targeting. Filtering of microarray expression data identified 20 potentially interesting genes consistently expressed in GSCs and consistently not expressed in NSCs. These preliminary data were then verified by experimental and bioinformatics means.

The present study identified a set of 20 genes that does not overlap with the 30 genes from our previous study [19]. In the latter investigation we aimed for genes highly up-regulated in GSCs with less consideration for their absolute expression levels in NSCs. In the current study we selected genes not expressed in NSCs and consistently expressed in GSCs.

Unlike many studies using brain tissue as a control, we used NSCs isolated from different areas of the adult human brain. Altogether we used 16 GSC and nine NSC cultures. To our knowledge, few reports have used such a high number of primary stem cell cultures to identify and verify candidate genes. While GSCs were propagated as spheres, the NSCs used for the validation experiments were cultured under three different sets of conditions, which reinforced the robustness of our findings. Both GSC cultures and the controls (NSC and NFC cultures) were properly matched with regard to state of differentiation and cell PDT (Figure 2). Although many of the selected 20 genes are involved in cell cycle or cell division, their expression was not altered in NSCs cultured in alternative growth conditions and with different cell proliferation rates (Supplementary Figures S3, S4 and S5).

To ensure a broader validity, we utilized microarrays, qPCR, immunolabeling, western blot and targeted proteomics in combination with public database mining and finally identified nine candidate genes (*PBK, CENPA, KIF15, DEPDC1, CDC6, DLG7, KIF18A, EZH2*, and *HMMR*) whose expression was confirmed using all experimental and bioinformatics verification methods (Table 1). Simultaneous up-regulation of these genes correlated negatively with patient survival in the *mesenchymal* GBM subtype (TCGA) (Figure 4C). Our multifaceted analysis further showed that the increased expression of the selected genes was characteristic of GSCs and not caused by variations in growth conditions, proliferation rates or differentiation state. It is also interesting that the set of the selected genes exhibited significantly higher expression levels in GBM compared to LGG, suggesting a dose-response relationship. *FNDC3B* was identified as a predictor of survival in LGG (Supplementary Figure S6). We found good correlation between mRNA and the corresponding protein expression for the selected 20 genes (*r* = 0.55 ± 0.41) (Table 1), which is of vital importance for downstream applications, such as gene silencing and pre-clinical testing of potential therapeutic targets, that rely on removal of not only the transcripts but also the corresponding proteins.

The TCGA research network classified GBMs into four molecular subtypes based on the tumor's gene expression patterns. Importantly, these subtypes were shown to have different responses to chemotherapy, thus emphasizing the clinical relevance of this classification [24]. Expression analysis of 200 GBM tissue samples from the TCGA database revealed that nine potential target genes were co-expressed in all GBM subtypes (Figure 3B). Previous studies have underlined the *proneural* characteristics of GSCs [28, 29]. Here we have shown that 11 out of the initial 20 selected genes, all highly up-regulated in GSCs, had an overall *proneural* character (Figure 3C). This is in keeping with results from previous studies [28, 29]. The suggested nine target genes were particularly up-regulated in the *proneural* GBM tissue samples, but were not limited to this group (Figure 3C). Furthermore, the majority of these were highly co-expressed at protein level with the notch ligand DLL3 (Figure 5B), which was previously identified as a *proneural* tumor marker [29]. Interestingly, the expression of these nine predominantly ‘*proneural*’ genes correlated with the survival of *mesenchymal* GBM patients, thus emphasizing the heterogeneity within the given GBM subtypes [30]. Alternatively, this could mean that *mesenchymal* GBM tumors that exhibit certain *proneural* features can be associated with worse prognosis.

Prior to the present study, six of the nine potential molecular targets (*EZH2, CDC6, PBK, KIF18A, HMMR* and *DLG7*) were shown to be up-regulated in GBM [31–35], thus underscoring the relevance of our study. One study identified *PBK, DLG7*, and *KIF18A* as up-regulated in GBM and classified them as mitosis, DNA replication, and chromosome (MRC) organization genes [34]. However, only the roles of *EZH2* and *HMMR* have been extensively studied in GBM and GSCs [31, 35]. *EZH2* has been identified as one of the key players in GBM and its increased expression correlates with a poor clinical outcome [31, 33]. *HMMR* has recently been suggested as a potential therapeutic target in GSCs [35]. *HMMR* is also involved in cell division [36] and is implicated in myelomas [37, 38] and breast cancer [39]. CDC6 binds BMI1, which is a known regulatory factor in GBM [40]. PBK, a serine/threonine kinase, is functionally related to the AKT pathway and can regulate cell cycle [41, 42]. Besides being a stem cell-related gene, *DLG7* also regulates KIF18A localization in the cell [43, 44]. The other genes have not previously been associated with GBM, although some have been linked to other cancers. For example centromere protein A (CENPA), is involved in cell division and is functionally related to several genes involved in GBM and cancer in general [34, 45]. DEP domain containing 1 protein (DEPDC1), has been implicated in bladder carcinogenesis [46] and in invasion/metastasis through the actions of p53 and p63 [47]. Recently, a cancer peptide vaccine has been used to target this protein [48]. Thus, it appears that almost all of the suggested nine target genes are directly or indirectly connected to cell cycle and/or cell division, in keeping with previous findings [19, 33, 34].

By querying the COGNOSCENTE database that visualizes known protein-protein interactions, we found that proteins encoded by 14 of our candidate genes, including the nine genes whose increased expression in GSCs has been confirmed by all methods (*PBK, CENPA, KIF15, DEPDC1, CDC6, DLG7, KIF18A, EZH2*, and *HMMR*) were all part of the same protein-protein interaction network (Figure 6). This analysis revealed that PBK, CENPA, CDC6, EZH2, MPP6 and MCC were highly interconnected and represented so called “hubs”. Until now only *EZH2* was recognized as a hub gene [33].

We have previously analyzed the expression of several signaling pathways known to be important in GSCs [19]. Many signaling pathways operate through protein modifications and are not always easily detected through mRNA analysis. Using targeted proteomics we calculated the degree of linear dependence (Pearson) between the expression levels of the proteins encoded by the 20 selected genes and the reporters of several cellular signaling pathways. This assembled proteins encoded by the selected genes and reporters of signaling pathways into three protein clusters. At the protein level, the increased expression of CENPA, DLG7, EZH2, KIF18A, DEPDC1, KIF15, NOL4 and FAM118A from protein cluster 1 positively correlated with the increment in levels of MTOR, DLL3 (Notch), PDGFB and STAT3. The same applied to protein cluster 2, where expression levels of PBK and RHBDD1 correlated with those of EGFR and PDGFR, and cluster 3 where expression levels of CDC6, MPP6, HMMR, FNDC3B, MDM1 and MCC correlated with those of SOX2 and NF1. Searching of the COGNOSCENTE database confirmed the results of targeted proteomics and further supported the notion that several proteins encoded by our candidate genes are functionally related to reporters of known signaling pathways in GBM. Due to the especially high number of common interactants (indicated in brackets), it is very probable that members of protein cluster 1: STAT3 and EZH2 (13), MTOR and CENPA (3) and MTOR and EZH2 (4), are functionally related. It has been shown previously that EZH2 binds to and methylates STAT3 in GSCs [49]. We also found that KIF18A and EZH2 shared common interactants with DLL3 and that DLG7, DEPDC1 and CENPA shared common interactants with STAT3. In cluster 2, PBK and RHBDD1 were both connected to EGFR through 11 and one common interactants, respectively. A link between PBK and EGFR has not been reported in GBM, although a functional relationship has been observed in other cancers [50]. In cluster 3 SOX2 and NF1 were connected to all four proteins CDC6, HMMR, MCC and MPP6 through shared interactants. The *NF1* gene encoding neurofibromin 1, is identified as a GBM suppressor gene that also defines the *mesenchymal* subtype of GBM [24]. It has recently been shown that the expression of HMMR correlated with the expression of the stemness marker SOX2 in GSCs [35]. Our results are in agreement with these studies. A search of the COGNOSCENTE database also revealed that CENPA both bound BMI1 and interacted with it indirectly through seven shared interactants. BMI1 bound CDC6, while HIF1A might be functionally related with EZH2, CDC6 and MPP6 with whom it shared seven, three and two common interactants respectively.

In addition to linking our candidate genes to reporters of signaling pathways and genes known to be important for GBM, we also identified several other genes that were highly interconnected within the network (Figure 6). UBC bound 11 of our proteins in addition to six of the reporters. The components of the ubiquitin-proteasome system (UPS) have already been evaluated as potential anti-cancer targets [51]. Another factor, NRF1, connected five proteins from our list (EZH2, HMMR, KIF18A, MPP6 and FAM118A) to one another and to DLL3 and NF1. It was previously reported that the expression of CDC6 was regulated by this transcription factor [52]. In addition to regulating expression of genes involved in mitochondrial function, NRF1 also binds a number of genes involved in cell cycle control [52]. This study further showed that NRF1 cooperates with E2F4 and other E2F family members to regulate expression of genes involved in cellular proliferation. Interestingly, PBK binds the transcription factor E2F4 which itself binds to a *cis* region of the *CDC6* gene (Figure 6).

We previously identified a 30-gene signature that was highly up-regulated in high-grade gliomas [19]. These findings were compared to the current set of selected genes. The cumulative querying of the COGNOSCENTE database using proteins from both lists showed several shared protein-protein interactions (Supplementary Figure S10). PBK bound CCNB1 while CENPA bound SHCBP1. ELAVL1 that bound RNA of seven genes from both lists seems to play a central role in this bigger network together with UBC, CDC6, E2F4, MYC and NRF1.

Global analysis of 134 microarrays showed that the GSCs and NSCs from this study clustered together with the GSCs and NSCs from other studies (Figure 8). Although all GSC cultures clustered together, the PCA could not separate clearly between GSCs, NSCs and BCC. However GSCs were separated from more differentiated cell types like neurons.

Our analysis showed that the sphere-forming ability and reciprocal values of cell PDT correlated best to each other and to levels of nestin, GFAP, TUBB3, the short variant of p-Ser727-STAT3 (unannotated), and Hedgehog and canonical Wnt signaling (Figure 6). It has been shown that *Shh* regulates the self-renewal of mouse stem cells [53], whereas Wnt signaling stimulates proliferation and suppresses differentiation of several types of stem cells including NSCs and GSCs [54, 55]. Our current results are in agreement with this and our previous findings [19].

We believe that the value of our study lies not only in the identification of nine important genes and potential therapeutic targets (*PBK, CENPA, KIF15, DEPDC1, CDC6, DLG7, KIF18A, EZH2*, and *HMMR*) in GSCs, but also in the range of independent methods used to verify the results. Gene knockdowns of EZH2 and HMMR already revealed that these two genes are essential for survival of GSCs and thus very promising new molecular targets for treatment of GBM [31, 35]. We are currently working on gene knockdowns of several genes from the presented list and have recently shown that targeting PBK/TOPK decreases growth and survival of glioma initiating cells *in vitro* and attenuates tumor growth *in vivo* [56]. Although the significance of the residual candidate genes remains to be determined, we clearly show that they are dysregulated in GSCs, important for patient survival and part of the same protein-protein network, which is shared with some of the principal genes that regulate GSC growth and tumorigenicity.

## MATERIALS AND METHODS

### Tumor specimens, primary tumor cultures, primary brain cultures and commercial cell lines

Following informed consent, tumor samples classified as GBM according to the World Health Organization criteria were obtained from patients undergoing surgical treatment at Oslo University Hospital in accordance with the appropriate Institutional Review Boards [57]. Normal brain tissue (SVZ, white matter and hippocampus) was harvested from human temporal lobes removed due to refractory epilepsy. The age, diagnosis and survival of the patients participating in this study can be found in Supplementary Table S3. Surgical biopsies were enzymatically and mechanically dissociated and Trypsin-EDTA (Gibco, Life Technologies, NYC, NY, USA) was added for enzymatic dissociation. Subsequently, 2 mg/ml human albumin (Octapharma pharmazeutika produktionges, Vienna, Austria) was used to block the Trypsin effect and the cells were washed in L-15 (Lonza, Basel, Switzerland) before being plated in serum-free neurosphere medium containing 10 ng/ml bFGF and 20 ng/ml EGF (both R&D Inc., Minneapolis, MN, USA), B27-supplement (1:50, Invitrogen, Carlsbad, CA, USA), 100 U/ml Penicillin/streptomycin (Lonza), 1 ng/ml Heparin (Leo Pharma, Ballerup, Denmark) and 8 mM Hepes (Lonza) in Dulbecco's modified essential medium with nutrient mix F-12 and Glutamax (DMEM/F12, Invitrogen). The cells were cultured in 75 cm^2^ non-treated flasks (Nunc, Roskilde, Denmark) at a density of 10^5^ cells/ml and supplemented with EGF and bFGF twice a week. GSC cultures were characterized for the following stemness markers: CD133, SSEA-1/CD15, CD44, CD166 and A2B5. GSC cultures were orthotopically xenografted to confirm tumor initiation properties [8, 56, 58, 59] (Mughal *et al*., in revision, Mughal *et al*., in prep).

NFCs (ReNcell VM Human Neural Progenitor Cell Line, SCC008, Merck Millipore, Darmstadt, Germany) were cultured as spheres in serum-free Neurobasal A medium (Gibco) containing B27 (Gibco), 2 mM L-glutamine, 10 ng/ml bFGF, and 20 ng/ml EGF (both from R&D Systems). Adherent NSCs were cultured in a modified neurosphere medium containing 1% FBS, 10 ng/ml bFGF and 20 ng/ml TGFα (*AD1%* medium) [22]. NSCs on RN were first incubated adherently in *AD1%* medium for one passage and were thereafter incubated on RN (Takara Bio, Otsu, Japan) in serum-free Neurobasal A medium (the same medium as for NFCs).

### RNA isolation and real-time quantitative reverse-transcription PCR (qPCR)

Total RNA was isolated using the RNeasy Mini Kit (Qiagen) and the concentration was determined with a Nanodrop spectrophotometer. For cDNA synthesis, experimental set up and oligonucleotide design, we used the procedure previously described [60]. cDNA was synthesized from 1 μg of RNA using a QuantiTect Reverse Transcription kit (Qiagen). qPCR was performed on an ABI PRISM 7900HT (Applied Biosystems, Life Technologies, Foster City, Ca, USA) using SYBR Premix Ex Taq™ (Takara, Otsu, Japan) or Taqman probes (Applied Biosystems) according to the manufacturer's protocol. A list of oligonucleotides used in this work can be found in Supplementary Table S4. Crossing point (CP) values (Supplementary Figure S1B) were generated using second-derivative calculation software (SDS2.2). Relative expression levels were calculated using 2^−ΔΔCT^ method and REST software [61]. Each GSC sample included at least three replicates (often different passages). The expression values for each gene were normalized to at least two house-keeping genes. The Relative Gene Expression values in GSCs (RE-GSC) for the 20 selected genes in Figure 1C were calculated using multiple controls (values obtained for all tested NSCs and NFCs) as reference. For expression analysis of FNDC3B we also used Taqman Probes: Hs00384650_m1 and Hs00981550 (Applied Biosystems).

### Western blot and targeted proteomics

Protein expression was analyzed in seven GSC and two to four NSC cultures. The cells were homogenized by triturating in Cell Extraction Buffer (Mammalian Cell Extraction Kit, Biovision, Milpitas, CA, USA) and centrifuged through a QIAshredder (Qiagen, Germantown, MD, USA). A total of 20–40 μg of whole protein extract was mixed with the loading buffer (NuPAGE; Life Technologies) and loaded onto a 4–12% gradient Nu-PAGE gel (Life Technologies). Protein gels were blotted onto 0.45-μm PVDF membranes. The membranes were blocked with 5% skimmed milk in TBS/0.1% Tween 20 (TBST; Life Technologies) and probed with primary antibodies diluted in the same solution. The primary antibodies, obtained from Cell Signaling Technologies (Danvers, MA, USA), were incubated in bovine serum albumin according to the recommended procedures. The secondary antibodies were HRP-conjugated anti-rabbit/mouse/goat/rat IgGs (1:10000). For a complete list of antibodies and the working concentrations, see Supplementary Table S5. The blots were developed using the Lumiglo Reserve CL Substrate kit (KLP, Gaithersburg, MD, USA), and detected using the Epi Chemi II Darkroom (Ultraviolet Laboratory Products, Upland, CA, USA). For targeted proteomics the relative protein expression (RPE) values were calculated as follows: the intensities of the protein bands from western blot were quantified using Adobe Photoshop (San Jose, CA, USA), background subtracted and normalized to the background subtracted intensities of the corresponding β-actin (ACTB) bands. To investigate the degree of linear dependence between the expression levels at the transcript and protein levels we compared the qPCR data to the quantified western data (RPE values) using the Pearson product-moment correlation coefficient (PPMCC *r*) calculation (Table 1). For hierarchical clustering of RPE values we used J-Express (Molmine, Bergen, Norway). This analysis was performed using Pearson correlation as a distance metric.

### Immunolabeling and confocal microscopy

GBM tissue samples were fixed in 4% PFA, cryoprotected in 20% sucrose and frozen in OCT (Tissue-TEK, Sakura Finetek, CA, USA). The blocks were cryo-sectioned and immunolabeling was performed as previously described [62, 63]. Cells were grown overnight on tissue-chamber slides (Nunc, Roskilde, Denmark) on RN (Takara) washed in PBS and fixed for 15 min in 4% PFA in PBS. The immunolabeling procedure was then performed following the procedure used for tissue sections. The complete list of antibodies can be found in Supplementary Table S6. Confocal images were acquired with an Olympus FV1000 confocal laser scanning microscope. To estimate the percentage of cells (Figure 2) 150–200 cells in at least five different microscope fields were counted using ImageJ (NIH, USA).

### Functional assays

#### Sphere-forming assay

Sphere-forming ability was measured by plating 500 cells/well in 200 μl neurosphere medium in 96-well plates. After 10 days, the plates were scanned using GelCount (Oxford-Optronix, Oxford, UK) and the sphere colonies were quantified using GelCount software.

#### Cell population doubling time (PDT)

We used the following formula: PDT = t log 2 / log Nt- log No where t = time period, Nt = number of cells at time t and No = initial number of cells.

### Bioinformatics and data mining

The initial selection of the 20 candidate genes was performed using excel-based analysis of 32875 transcripts from the array data set GSE31262 [19]. By filtering the log-transformed gene expression data, we selected 20 genes whose RNA expression levels on the logarithmic scale were below zero in the NSC cultures and above zero in GSC cultures.

REMBRANDT: Microarray data from the Repository for Molecular Brain Neoplasia Data (National Cancer Institute. 2005. REMBRANDT home page http://rembrandt.nci.nih.gov were accessed on May the 15th 2012. The repository contained 249 microarrays of GBM grade IV patients and 66 microarrays of Astrocytoma grade II patients. The microarrays were from the Affymetrix Gene Chip Human Genome U133 Plus 2.0 Array platform. The R/Bioconductor package Robust Multiarray Average (RMA) [64, 65] was used for pre-processing of the data. The selected 20 genes were represented by 48 different probes in the Affymetrix arrays. For each probe we fitted an univariate logistic regression model, with GBM/low-grade as (a dichotomous) response and the gene expression values as explanatory variables. *P*-values from the logistic regression were corrected for multiple testing using the Benjamini-Hochberg procedure [66].

TCGA: The gene expression data (https://tcga-data.nci.nih.gov/tcga/tcgaHome2.jsp) are based on tissue samples from 598 GBMs and 10 normal samples (Supplementary Figure S6C). The results obtained for GBM could be published without restrictions. The Cox proportional hazards model was used to correlate gene expression data to survival. Both univariate (individual genes) and multivariate models (several genes) were investigated. Based on a fitted Cox model, patients were divided into either a good or a bad prognosis group. Differences in (actual) survival between the prognostic groups were evaluated in terms of a log rank test.

For Figure 4: Survival/expression data (200 GBMs) [24] were downloaded from https://tcga-data.nci.nih.gov/docs/publications/gbm_exp/ and processed using J-Express (Molmine) for hierarchical clustering. Patient survival was calculated using Prism 6 (GraphPad, La Jolla, CA, USA).

For Figure 6: For construction of the protein interaction networks we used the COGNOSCENTE database (http://vanburenlab.tamhsc.edu/cognoscente.html) that enables visualization of biomolecular interaction documented in the literature. The gene *P2RY5* was not in the database while there were no interactions found for *FILIP1L*. Due to complexity of the network only the protein-protein interactions concerning the 20 selected proteins, BMI1 and HIF1A are presented (Figure 6 and Supplementary Figure S8). Some of the additional protein-protein interactions discussed in the text that are not included in Supplementary Figures S8–S10 can be easily reproduced by querying the COGNOSCENTE database.

For Figure 8: PCA analysis was calculated using R, version 3.1.2. The following sets of microarrays were downloaded from the GEO database, quantile normalized and used for this analysis: NSC and GSC cultures and GBM tissues from: H91, H95, H80, NFCs, T65, T08, TC3, TC4, T96, T11 and T59 (encompassed in the GEO sets GSE60705, GSE53800 and GSE41467) in addition to iPS, neurons, iPS-derived neurons, astrocytes, fibroblasts, NSCs, NFCs, breast cancer cells (BCC, cancer stem cells and cell lines), leucocytes, brain tissue, GBM, ESCs, gliomas cell lines and many additional sets of GSCs [encompassed in the GEO sets GSE41468, GSE34987, GSE36426 (GSCs), GSE42133, GSE43364, GSE43452, GSE43903, GSE47515, GSE42265, GSE32658, GSE37077, GSE41565 and GSE36102]. NSC cultures from patients: H91, H95, H80 were represented by samples from HPC, SVZ, WM and GM and were grown either as spheres or in *AD1%* medium.

## SUPPLEMENTARY FIGURES AND TABLES


