# Supplementary material for: Combined expressional analysis, bioinformatics and targeted proteomics identify new potential therapeutic targets in glioblastoma stem cells
Source: Oncotarget. 2015 Jul 20;6(28):26192–215. doi: 10.18632/oncotarget.4613 (PMC4694895; doi:10.18632/oncotarget.4613)
Supplement: Supplementary file 1 [file oncotarget-06-26192-s001.pdf]

SUPPLEMENTARY FIGURES AND TABLES

A

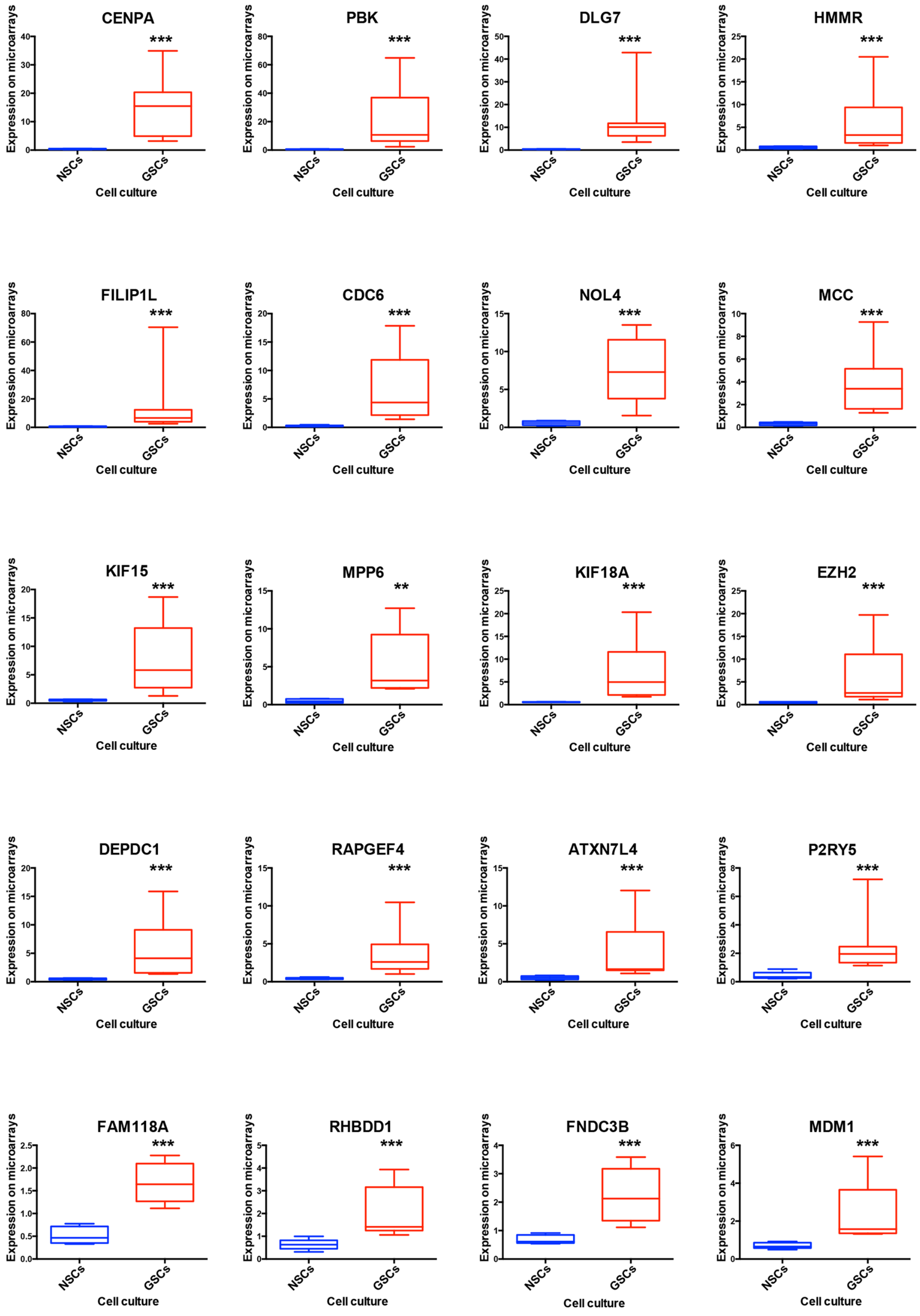

Supplementary Figure S1: A. Expression of the 20 selected genes on the microarray. Box- and-Whisker Plots of microarray data were calculated using NSC (n = 5) and GSC (n = 9) cultures. The bottom and top of the box indicate the 25th and 75th percentile (the lower and upper quartiles, respectively), and the band near the middle of the box is the 50th percentile (the median). The ends of the whiskers represent the minimum and maximum of all data. Asterisks correspond to p values and indicate level of significance: **=($p \approx 0.001–0.01$) and ***=($p \approx 0.0001– 0.001$). (*Continued*)

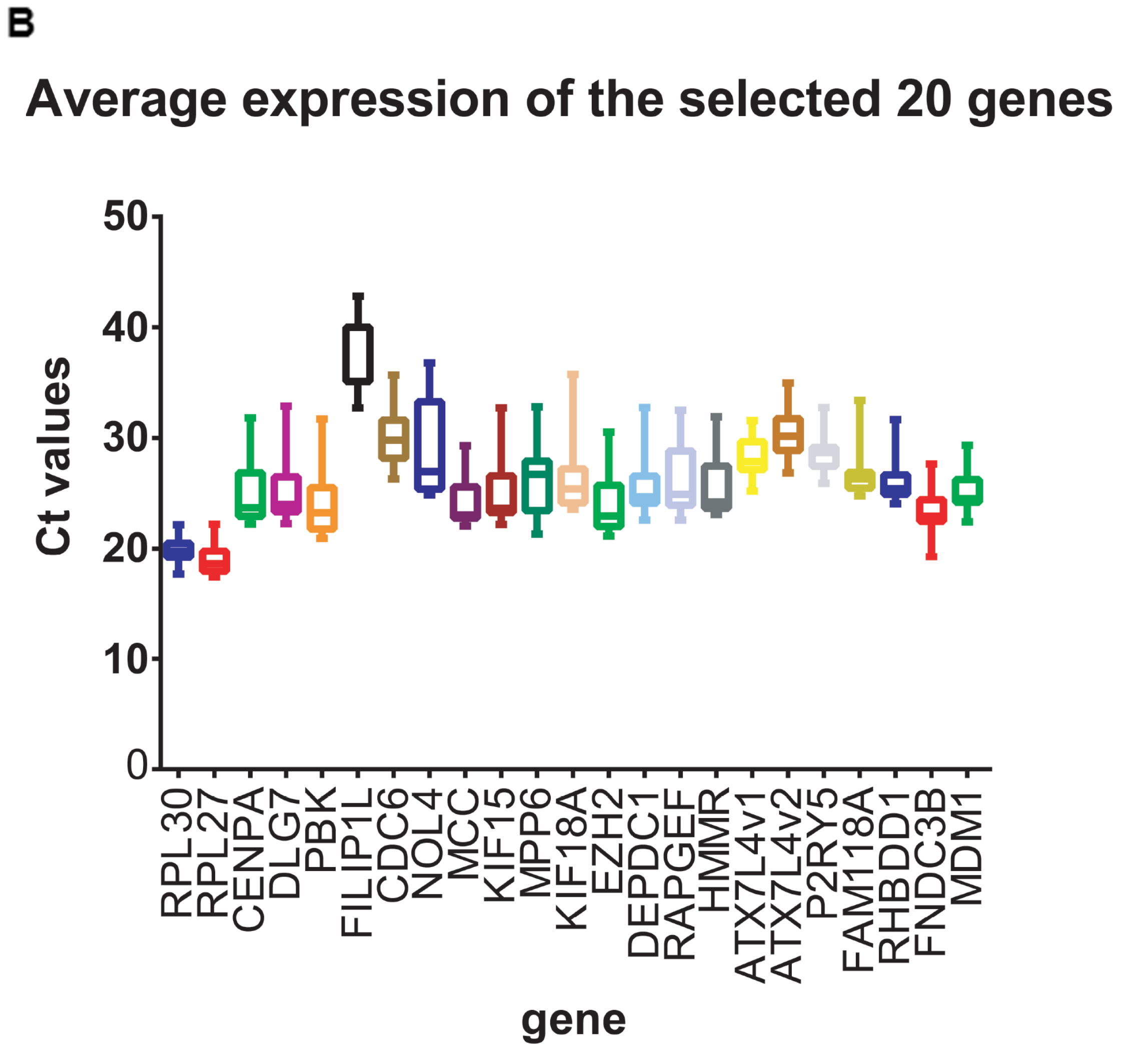

Supplementary Figure S1: (*Continued*) B. The average expression of the 20 selected genes in qPCR is presented as Ct values.

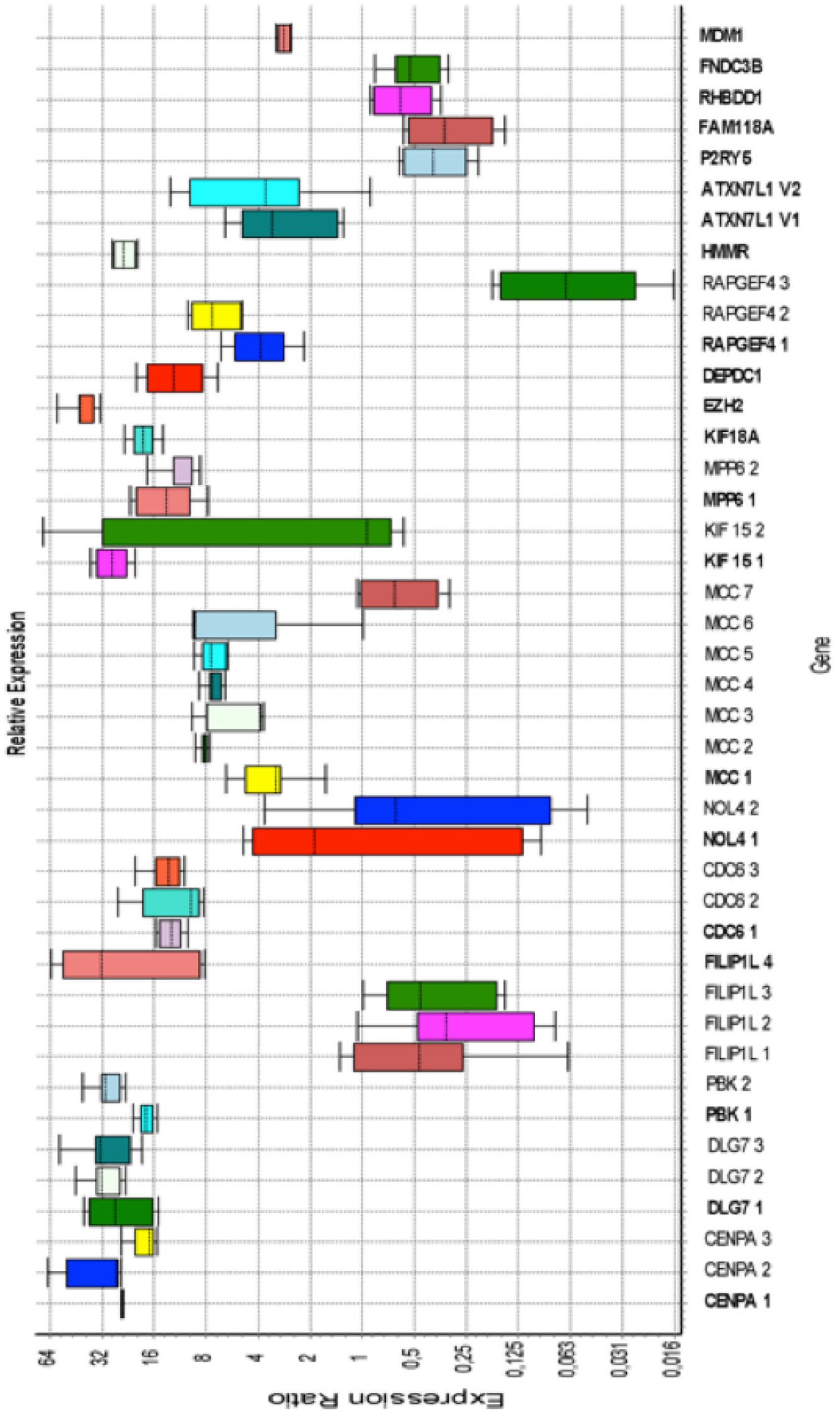

Test for best performing oligonucleotides. Expression of the 20 selected genes was calculated for three GSC and two NSC cultures. One to seven sets of oligonucleotides were tested for each gene. Best performing oligonucleotides were chosen for further analysis (bold).

	Gene	Expression	Std. Error	95% C.I.	P(H1)	Result
	RPL30	1.054				
	RPL27	0.949				
1	CENPA 1	24.568	24.274 – 24.874	24.068 – 24.983	0	UP
1	CENPA 2	34.606	24.717 – 61.934	24.717 – 64.927	0	UP
1	CENPA 3	18.28	15.305 – 22.682	15.305 - 24.383	0	UP
2	DLG7 1	24.838	15.675 – 39.389	15.204 - 40.577	0	UP
2	DLG7 2	31.068	23.292 - 37.002	23.292 - 43.878	0	UP
2	DLG7 3	30.253	18.930 - 39.695	18.930 - 53.896	0	UP
3	PBK 1	17.814	15.496 - 19.803	15.353 - 21.000	0	UP
3	PBK 2	29.746	23.185 - 34.391	23.185 - 40.199	0.042	UP
4	FILIP1L-1	0.41	0.162 - 1.349	0.080 - 1.349	0.476	
4	FILIP1L-2	0.251	0.077 - 0.605	0.077 - 0.994	0.107	
4	FILIP1L-3	0.366	0.150 - 0.778	0.150 - 0.955	0	DOWN
4	FILIP1L-4	22.196	8.171 - 56.629	8.171 - 62.127	0	UP
5	CDC6 1	12.713	10.861 - 15.355	10.295 - 15.489	0.042	UP
5	CDC6 2	12.446	8.238 - 22.657	8.238 - 25.481	0	UP
5	CDC6 3	13.759	10.694 - 17.419	10.694 - 20.022	0	UP
6	NOL4 1	0.687	0.110 - 4.658	0.095 - 4.825	0.603	
6	NOL4 2	0.352	0.050 - 1.651	0.050 - 3.394	0.613	
7	MCC 1	3.404	2.647 - 5.492	1.784 - 6.074	0	UP
7	MCC 2	8.216	7.636 - 8.697	7.636 - 9.110	0	UP
7	MCC 3	5.201	3.773 - 9.406	3.773 - 9.649	0	UP
7	MCC 4	7.239	6.237 - 7.950	6.237 - 8.626	0	UP
7	MCC 5	7.345	5.936 - 8.720	5.936 - 9.268	0	UP
7	MCC 6	4.452	1.020 - 9.538	0.994 - 9.538	0.181	
7	MCC 7	0.59	0.347 - 1.064	0.319 - 1.064	0.195	
8	KIF 15 1	27.927	21.989 - 35.971	20.866 - 37.170	0	UP
8	KIF 15 2	3.091	0.573 - 48.210	0.573 - 66.594	0.894	
9	MPP6 1	13.554	9.413 - 21.853	8.030 - 21.854	0	UP
9	MPP6 2	11.709	8.716 - 13.629	8.716 - 16.945	0	UP
10	KIF18A	18.44	15.487 - 22.291	14.453 - 23.402	0	UP
11	EZH2	41.415	32.694 - 46.833	32.694 - 56.169	0	UP
12	DEPDC1	11.967	7.774 - 19.076	7.008 - 20.174	0	UP
13	RAPGEF4 1	3.845	2.612 - 5.975	2.254 - 6.433	0	UP
13	RAPGEF4 2	7.044	4.933 - 9.856	4.933 - 10.153	0	UP
13	RAPGEF4 3	0.057	0.024 - 0.177	0.017 - 0.177	0	DOWN
14	RHAMM	23.759	20.169 - 27.846	20.024 - 28.257	0	UP
15	ATXN7L1 V1	2.743	1.297 - 5.262	1.296 - 6.096	0.033	UP
15	ATXN7L1 V2	3.9	1.920 - 12.078	1.058 - 12.858	0.181	
16	P2RY5	0.369	0.239 - 0.605	0.218 - 0.610	0	DOWN
17	FAM118A	0.299	0.167 - 0.570	0.151 - 0.576	0	DOWN
18	RHBDD1	0.569	0.379 - 0.898	0.353 - 0.898	0.042	DOWN
19	FNDC3B	0.497	0.320 - 0.691	0.320 - 0.817	0	DOWN
20	MDM1	2.849	2.597 - 3.140	2.578 - 3.140	0	UP

Supplementary Figure S2: Test for best performing set of oligonucleotides with statistics.

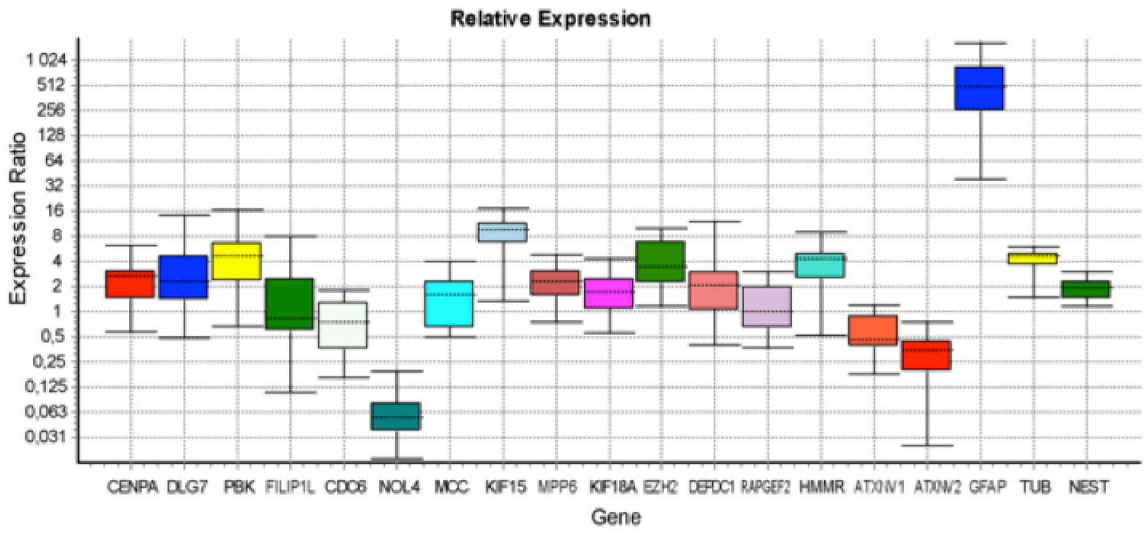
Relative Expression
Expression Ratio
1 024
512
256
128
64
32
16
8
4
2
1
0,5
0,25
0,125
0,063
0,031
CENPA
DLG7
PBK
FILIP1L
CDC6
NOL4
MCC
KIF15
MPP6
KIF18A
EZH2
DEPDC1
RAPGEF2
HMMR
ATXN1
ATXN2
GFAP
TUB
NEST
Gene

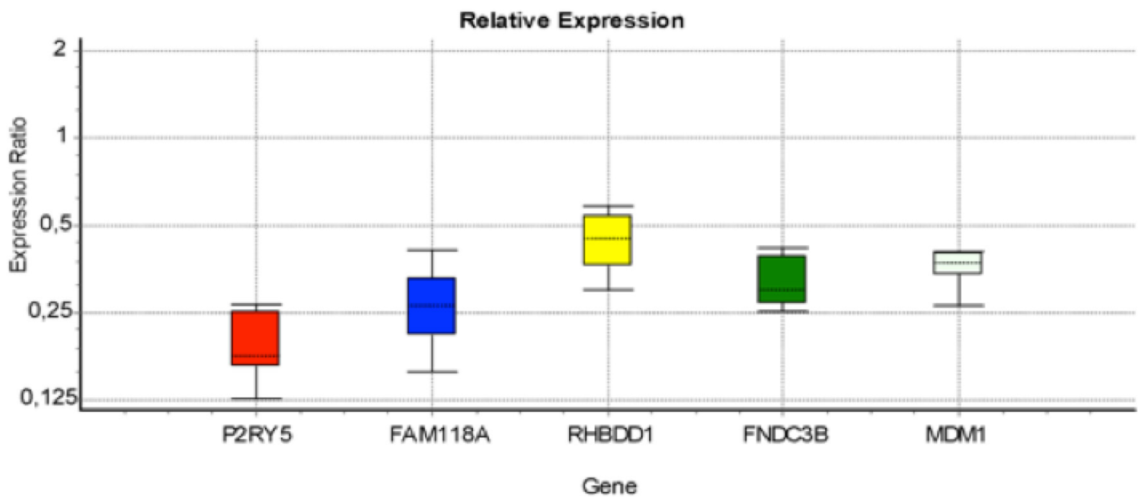
Relative Expression
Expression Ratio
2
1
0,5
0,25
0,125
P2RY5
FAM118A
RHBDD1
FNDC3B
MDM1
Gene

	Gene	Expression	Std. Error	95% C.I.	P(H1)	Result
ref	RPL30	0.991				
ref	RPL27	1.009				
1	CENPA	2.175	1.244 - 3.822	0.706 - 5.860	0.117	
2	DLG7	2.423	0.740 - 9.379	0.513 - 13.494	0.284	
3	PBK	3.86	1.459 - 8.245	0.796 - 15.203	0.057	
4	FILIP1L	1.01	0.421 - 6.573	0.123 - 7.868	0.937	
5	CDC6	0.643	0.290 - 1.459	0.161 - 1.803	0.539	
6	NOL4	0.054	0.028 - 0.107	0.019 - 0.177	0.007	DOWN
7	MCC	1.379	0.591 - 2.838	0.522 - 3.903	0.426	
8	KIF15	7.051	2.182 - 14.404	1.425 - 16.842	0.035	UP
9	MPP6	2.183	1.328 - 3.484	0.906 - 4.613	0.063	
10	KIF18A	1.661	0.942 - 2.777	0.664 - 4.027	0.192	
11	EZH2	3.604	1.784 - 8.238	1.251 - 9.467	0.027	UP
12	DEPDC1	2.045	0.923 - 6.260	0.516 - 11.360	0.325	
13	RAPGEF4	1.063	0.550 - 2.406	0.377 - 2.867	0.882	
14	HMMR	2.965	0.892 - 6.087	0.536 - 8.722	0.077	
15	ATXNV1	0.509	0.309 - 1.033	0.189 - 1.181	0.252	
15	ATXNV2	0.22	0.037 - 0.545	0.027 - 0.734	0.035	DOWN
16	P2RY5	0.19	0.149 - 0.259	0.130 - 0.265	0.028	DOWN
17	FAM118A	0.259	0.189 - 0.360	0.160 - 0.401	0.007	DOWN
18	RHBDD1	0.434	0.339 - 0.557	0.307 - 0.577	0.022	DOWN
19	FNDC3B	0.32	0.262 - 0.403	0.253 - 0.417	0	DOWN
20	MDM1	0.352	0.311 - 0.406	0.271 - 0.406	0.087	
	GFAP	345.288	53.839 - 1 141.827	39.262 - 1 560.456	0.006	UP
	TUB	3.787	1.869 - 5.397	1.578 - 5.776	0.027	UP
	NEST	1.894	1.337 - 2.571	1.204 - 2.962	0.03	UP

Supplementary Figure S3: Relative expression of the selected 20 genes, *NES*, *GFAP*, and *TUBB3* in NFCs. The NSC cultures grown on retronectin were used as controls. NFCs expressed more *TUBB3* and *NES* than NSCs, while the expression of *GFAP* was more than 300 fold higher in NFCs. The expression of two genes (*KIF15* and *EZH2*) was higher in NFCs while NOL4, *ATXNV2, P2RY5, FAM118A, RHBDD1* and *FNDC3B* were down regulated in NFCs. NSC = human adult neural stem cells NFC = human neural fetal cells.

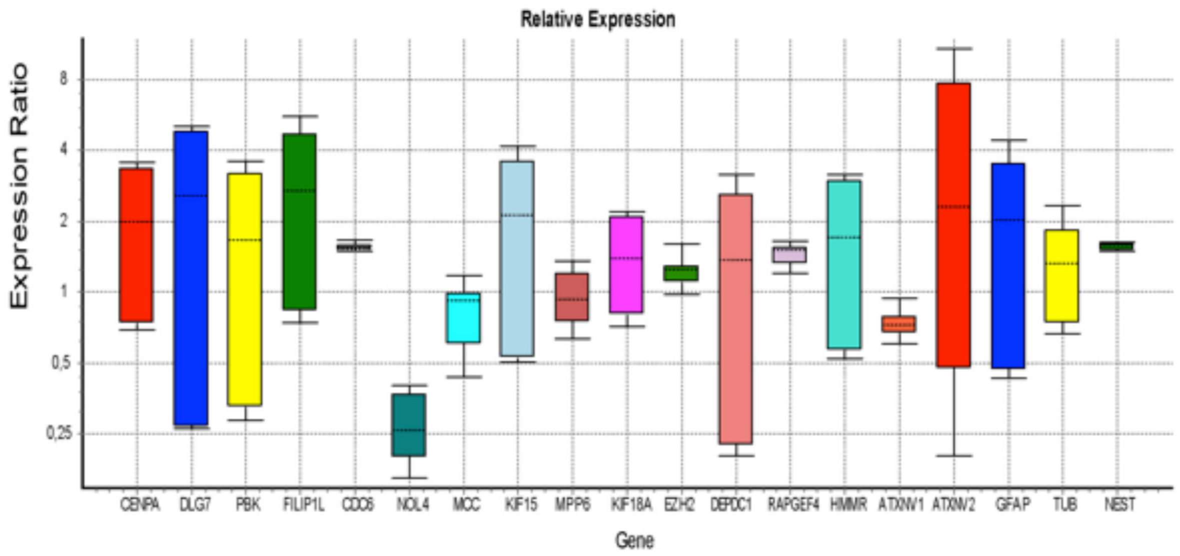
Relative Expression
Expression Ratio
8
4
2
1
0,5
0,25
CENPA
DLG7
PBK
FILIP1L
CDC6
NOL4
MCC
KIF15
MPP6
KIF18A
EZH2
DEPDC1
RAPGEF4
HMMR
ATXNV1
ATXNV2
GFAP
TUB
NEST
Gene

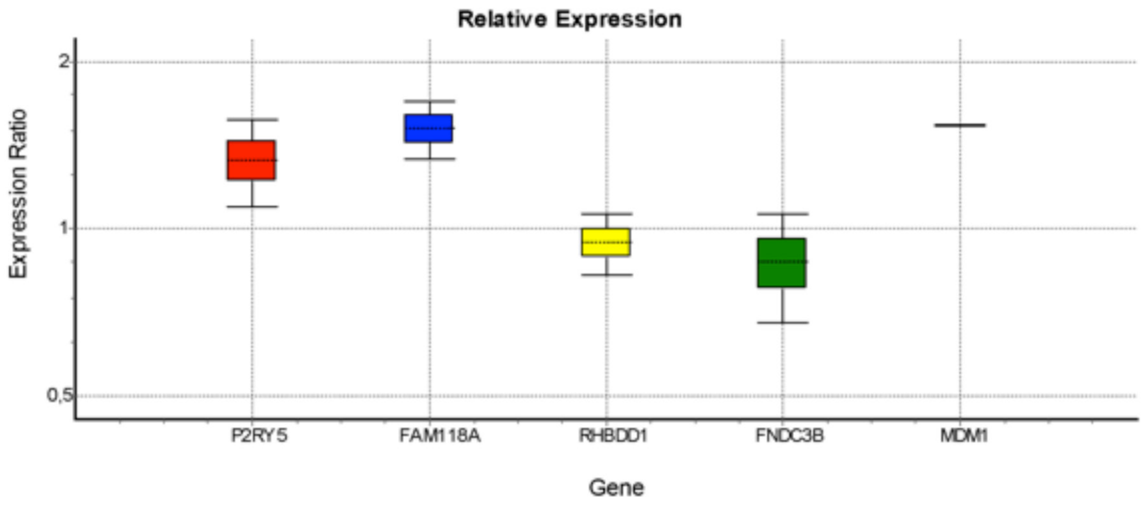
Relative Expression
Expression Ratio
2
1
0,5
P2RY5
FAM118A
RHBDD1
FNDC3B
MDM1
Gene

	Gene	Expression	Std. Error	95% C.I.	P(H1)	Result
ref	RPL30	0.979				
ref	RPL27	1.022				
1	CENPA	1.575	0.724 - 3.484	0.695 - 3.543	0.607	
2	DLG7	1.15	0.266 - 4.922	0.264 - 5.035	0.607	
3	PBK	1.016	0.309 - 3.363	0.290 - 3.561	0.805	
4	FILIP1L	2.015	0.794 - 4.982	0.753 - 5.473	0.41	
5	CDC6	1.558	1.495 - 1.645	1.495 - 1.669	0.03	UP
6	NOL4	0.262	0.191 - 0.393	0.167 - 0.399	0.03	DOWN
7	MCC	0.782	0.503 - 1.045	0.448 - 1.154	0.753	
8	KIF15	1.418	0.503 - 3.774	0.503 - 4.112	0.607	
9	MPP6	0.938	0.716 - 1.276	0.645 - 1.345	0.831	
10	KIF18A	1.274	0.777 - 2.180	0.728 - 2.191	0.607	
11	EZH2	1.228	1.047 - 1.375	0.993 - 1.569	0.201	
12	DEPDC1	0.784	0.213 - 2.771	0.203 - 3.092	0.731	
13	RAPGEF4	1.445	1.260 - 1.595	1.210 - 1.630	0.094	
14	HMMR	1.291	0.550 - 3.079	0.525 - 3.151	0.607	
15	ATXNV1	0.738	0.646 - 0.843	0.612 - 0.926	0.13	
15	ATXNV2	1.646	0.403 - 9.463	0.234 - 10.612	0.701	
16	P2RY5	1.312	1.171 - 1.495	1.107 - 1.559	0.335	
17	FAM118A	1.51	1.398 - 1.644	1.349 - 1.692	0	UP
18	RHBDD1	0.939	0.864 - 1.029	0.832 - 1.061	0.66	
19	FNDC3B	0.851	0.741 - 1.004	0.690 - 1.056	0.66	
20	MDM1	1.54	1.539 - 1.541	1.538 - 1.541	0	UP
	GFAP	1.337	0.439 - 3.751	0.432 - 4.311	0.642	
	TUBB3	1.213	0.691 - 1.981	0.669 - 2.280	0.707	
	NEST	1.568	1.497 - 1.630	1.493 - 1.635	0	UP

Supplementary Figure S4: Relative expression of the 20 selected genes *NES*, *GFAP*, and *TUBB3* in NSCs cultured as spheres. The NSC cultures grown on retronectin were used as controls. The expression levels of *GFAP* and *TUBB3* were similar between the NSCs cultured as spheres and on retronectin, while *NES* was slightly (1.6x) up-regulated in spheres. The expression of 20 genes was only mildly influenced by growth conditions. *CDC6*, *FAM118A* and *MDM1* were somewhat up-regulated (1.5x) in spheres. The relative expression of *NOL4* was lower in spheres then on retronectin.

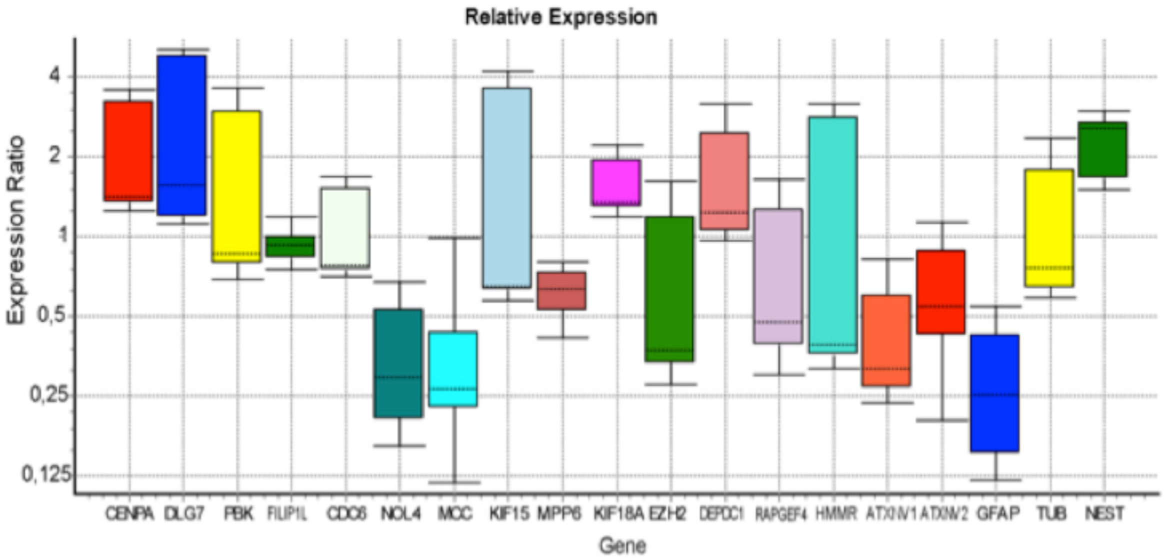

Relative Expression
Expression Ratio
4
2
1
0,5
0,25
0,125
CENPA
DLG7
PBK
FILIP1L
CDC6
NOL4
MCC
KIF15
MPP6
KIF18A
EZH2
DEPDC1
RAPGEF4
HMMR
ATXN1
ATXN2
GFAP
TUB
NEST
Gene

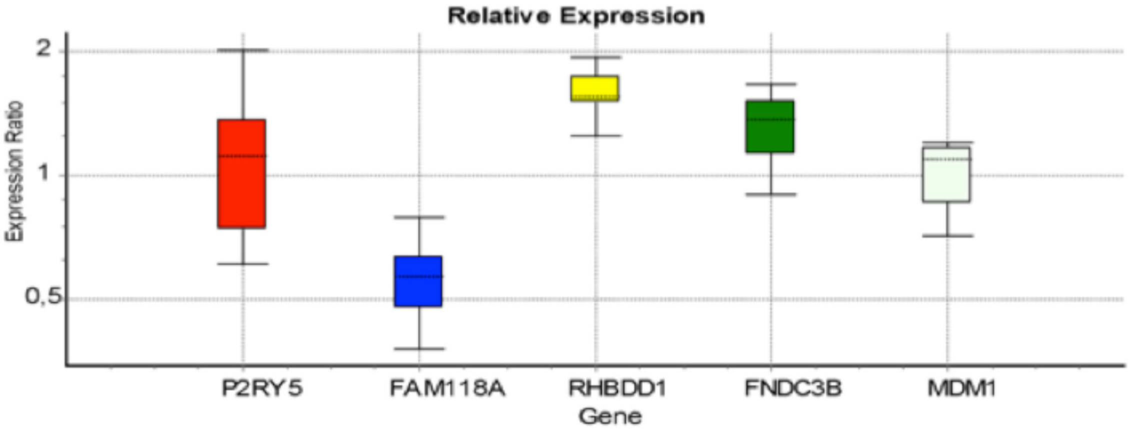

Relative Expression
Expression Ratio
2
1
0,5
P2RY5
FAM118A
RHBDD1
FNDC3B
MDM1
Gene

	Gene	Expression	Std. Error	95% C.I.	P(H1)	Result
ref	RPL30	0.978				
ref	RPL27	1.023				
1	CENPA	1.839	1.323 - 3.411	1.262 - 3.536	0.062	
2	DLG7	2.06	1.139 - 4.874	1.120 - 5.023	0.046	UP
3	PBK	1.285	0.761 - 3.221	0.698 - 3.539	0.967	
4	FILIP1L	0.92	0.808 - 1.026	0.757 - 1.155	0.436	
5	CDC6	0.955	0.715 - 1.528	0.707 - 1.645	0.897	
6	NOL4	0.316	0.199 - 0.620	0.169 - 0.666	0.022	DOWN
7	MCC	0.301	0.151 - 0.739	0.118 - 0.961	0.05	DOWN
8	KIF15	1.138	0.583 - 3.670	0.568 - 4.075	0.959	
9	MPP6	0.618	0.496 - 0.761	0.430 - 0.792	0.032	DOWN
10	KIF18A	1.514	1.240 - 2.122	1.186 - 2.190	0.041	UP
11	EZH2	0.541	0.306 - 1.270	0.279 - 1.544	0.297	
12	DEPDC1	1.469	0.986 - 2.621	0.965 - 3.056	0.215	
13	RAPGEF4	0.616	0.376 - 1.500	0.316 - 1.626	0.314	
14	HMMR	0.73	0.341 - 3.002	0.320 - 3.143	0.815	
15	ATXNV1	0.381	0.257 - 0.643	0.237 - 0.786	0.032	DOWN
15	ATXNV2	0.556	0.359 - 0.936	0.229 - 1.095	0.134	
16	P2RY5	1.057	0.654 - 1.528	0.615 - 1.936	0.931	
17	FAM118A	0.554	0.449 - 0.676	0.390 - 0.775	0.096	
18	RHBDD1	1.582	1.443 - 1.836	1.274 - 1.917	0.031	UP
19	FNDC3B	1.291	1.031 - 1.587	0.919 - 1.656	0.266	
20	MDM1	0.994	0.766 - 1.201	0.720 - 1.203	0.901	
	GFAP	0.254	0.131 - 0.439	0.120 - 0.524	0.032	DOWN
	TUB	0.969	0.618 - 1.866	0.587 - 2.247	0.964	
	NEST	2.245	1.599 - 2.814	1.516 - 2.929	0.062	

Supplementary Figure S5: Relative expression of the selected 20 genes, *NES*, *GFAP*, *TUBB3* in NSCs cultured in *AD1%* medium. The NSCs cultured on retronectin were used as controls. None of the 20 candidate genes were highly up-regulated. *DLG7, KIF18A* and *RHBDD1* were 1.5-2 fold up-regulated while *NOL4, MCC, MPP6* and *ATXN* (variant 1) were down regulated in cells in *AD1%* medium.

A

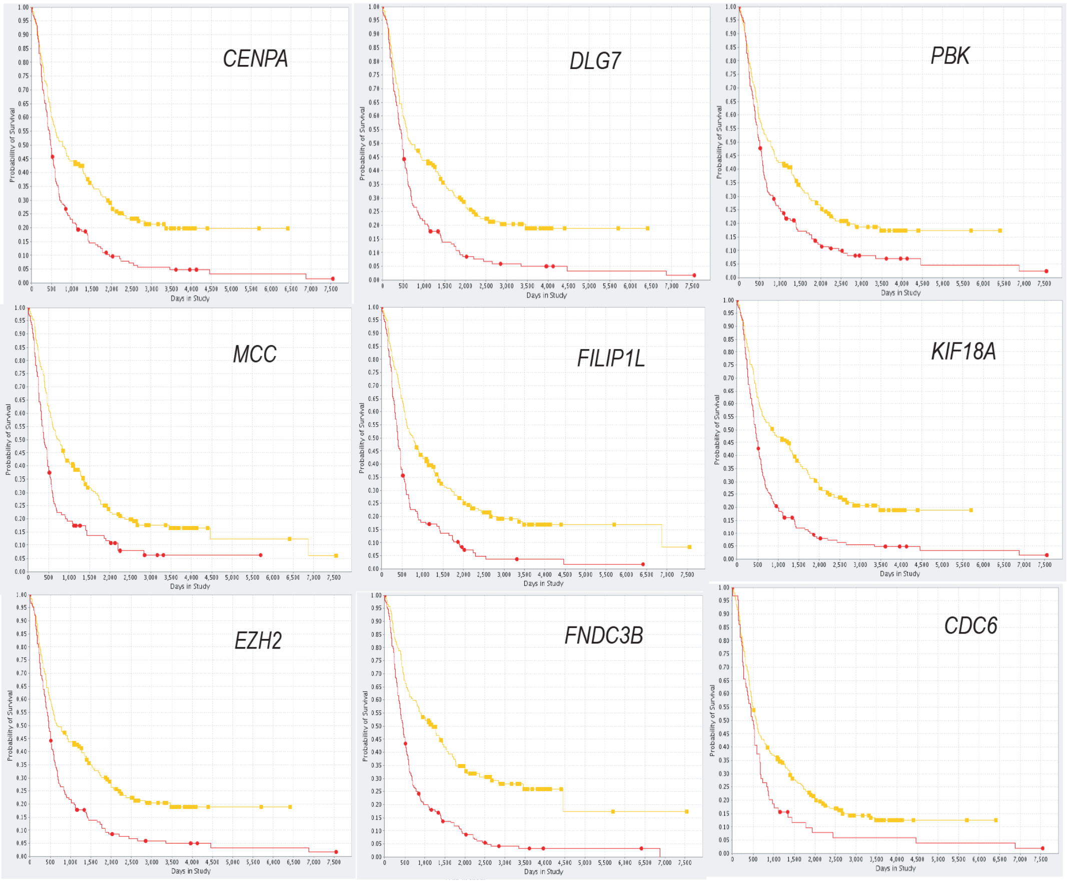

B

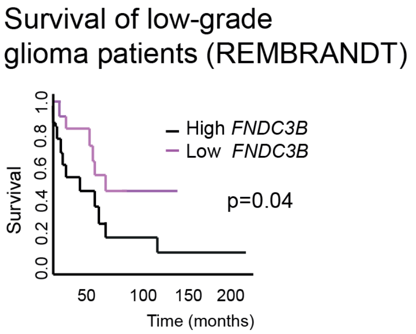

C

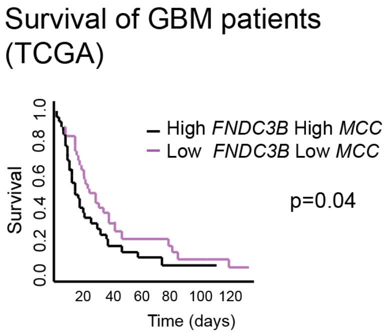

Supplementary Figure S6: A–C. Kaplan-Meier survival plots (K-M) for the expression of the 20 selected genes (A). A K-M displays the survival rate at each time point for samples with certain expression characteristics. K-M plots calculated for the group of “all gliomas” (from REMBRANDT) show that the expression levels of 15 genes were predictive of survival. Only plots for CENPA (p = 3.93E-06), DLG7 (p = 5.79E-06), PBK (p = 5.00E-04), MCC (p = 2.70E-06); FILIP1L (p = 3.14E-08), KIF18A (4.95E-08), EZH2 (6.24E-05), FNDC3B (p = 0) and CDC6 (p = 0.0104) are shown here. The red line indicates the survival for the patients with up-regulated expression of a particular gene. The yellow line represents patients with intermediate survival. The p values that describe the significance of difference of survival between up-regulated and intermediate group of samples for a specific threshold are shown in the Table below. (B–C) Correlation between gene expression and survival was calculated using REMBRANDT (B) and TCGA (C) database. (B) K-M plot for the LGG group showing that high expression of *FNDC3B* correlated negatively with survival. Pink line corresponds to the survival of the patients with low expression of *FNDC3B*. Black line indicates the survival of the patients with high levels of *FNDC3B* expression (log- rank test $p = 0.04$). (C) K-M plot for GBM patients group showing that the coinciding high expression of two genes *FNDC3B* and *MCC* correlated negatively with patient survival. Survival for the group with low expression levels of *MCC* and *FNDC3B* is indicated as a pink line. Black line specifies survival in the group with high expression of *MCC* and *FNDC3B* (log-rank test $p = 0.04$). (*Continued*)

		Log-Rank-P-value (Upreg. VS. Intermediate)				
		All Gliomas	Significance	GBM	Significance	Fold up-regulation
1	CENPA	3.93E-06	*	0.584		4
2	DLG7	5.79E-06	*	0.387		6
3	PBK	5.00E-04	*	0.836		10
4	FILIP1L	3.14E-08	*	0.0391	*	2
5	CDC6	0.0104	*	0.0150		2
6	NOL4	NA		NA		2
7	MCC	2.70E-06	*	0.0447	*	2
8	KIF15	0.00176	*	0.170		3
9	MPP6	0.0332	*	0.702		2
10	KIF18A	4.95E-08	*	0.268		5
11	EZH2	6.24E-05	*	0.715		8
12	DEPDC1	5.92E-06	*	0.195		3
13	RAPGEF4	NA		NA		2
14	HMMR	2.25E-04	*	0.622		2
15	ATXN7L4|ATXN7L1	NA		NA		2
16	P2RY5	0.0148	*	0.949		2
17	FAM118a	0.0293	*	0.167		3
18	RHBDD1	0.483		0.881		2
19	FNDC3B	0	*	0.0361	*	2
20	MDM1	0.302		0.477		2

Supplementary Figure S6: (*Continued*) D. Statistical parameters of Kaplan-Meier survival data for all 20 genes (REMBRANDT data-base). Log-rank-*p*-values that tell the significance of difference of survival between "up-regulated" and "intermediate" groups of samples for the specific fold upregulation threshold are indicated. The thresholds values (same as fold upregulation) are chosen to give the best separation. Fifteen out of 20 selected genes can be used as predictors of patient survival in gliomas while 3 were predictive of survival in GBM.

Additional western data

A

		**Western quantification (protein)**					**Correlation RNA/protein**
No.	**GENE ID**	Expected size (kDa)	Observed size (kDa)	Fold change	Std. Error	95% C.I.	correlation
1	**CENPA**	16	17	8.01	1.87	3.74	**0.76**
2	**DLG7**	95, 85	119	41.04	6.02	12.04	**0.70**
3	**PBK**	36, 50	38	24.59	5.73	11.45	**0.74**
4	**FILIP1L**	130, 102	108	31.68	3.50	7.00	0.33
4	**FILIP1L**	100, 81	92	0.34	0.10	0.20	0.13
4	**FILIP1L**		58	6.68	1.78	3.56	0.33
4	**FILIP1L**	30	32	2.24	0.52	1.03	**0.73**
5	**CDC6**	63	65	8.79	1.36	2.71	**0.66**
6	**NOL4**	71, 59	70	7.14	0.60	1.20	**0.70**
7	**MCC**	93, 113	125	0.32	0.05	0.11	**-0.42**
8	**KIF15**	150	170	4.67	0.64	1.29	**0.73**
9	**MPP6**	61	57	4.62	0.97	1.93	**0.80**
10	**KIF18A**	102	100	22.39	5.91	11.81	**0.89**
11	**EZH2**	80	85	17.14	4.75	9.51	**0.92**
12	**DEPDC1**	93, 64	113, 65	49.95	19.29	38.59	**0.52**
13	**RAPGEF4**	99	100	0.18	0.03	0.06	**-0.59**
14	**HMMR**	84	95	7.69	1.92	3.83	**0.86**
15	**ATXN7L4**	92, 78, 16	40	0.87	0.12	0.23	**0.40**
16	**P2RY5**	39	35	0.14	0.04	0.09	**0.52**
17	**FAM118A**	40.3	48	7.34	2.09	4.19	-0.36
17	**FAM118A**		60	0.55	0.15	0.30	**0.61**
18	**RHBDD1**	36	43	2.33	0.84	1.69	**-0.05**
19	**FNDC3B**	133	130	17.52	3.65	7.30	**0.63**
20	**MDM1**	80	120	30.40	5.18	10.37	0.54
20	**MDM1**		230				**0.88**
						AVERAGE	0.55

B

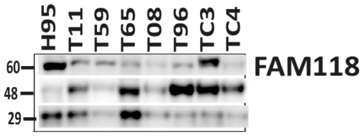

Supplementary Figure S7: Results of western blot analysis. A. Quantification of western blot with statistical parameters. Fold change values were calculated using expression values for each protein band. These expression values were calculated using Photoshop (Adobe). All values were back-ground subtracted and normalized to actin prior to calculation. Correlation (Pearson) was calculated in excel using quantified western data and the mRNA expression values (qPCR). **B.** Using Anti-FAM118A antibody we detected three protein bands. The band of the expected size (48 kDa) was up-regulated in GSCs while the two additional bands were down-regulated in GSCs (60 and 29 kDa). Expression values for the band of 60 kDa correlated well with the RNA expression indicating that this might be the band that corresponds to the mRNA detected with qPCR (Figure 1). Anti-FILIP1L antibody detected two bands close to the expected size (92 and 32 kDa) and one new band of 58 kDa (Figure 5).

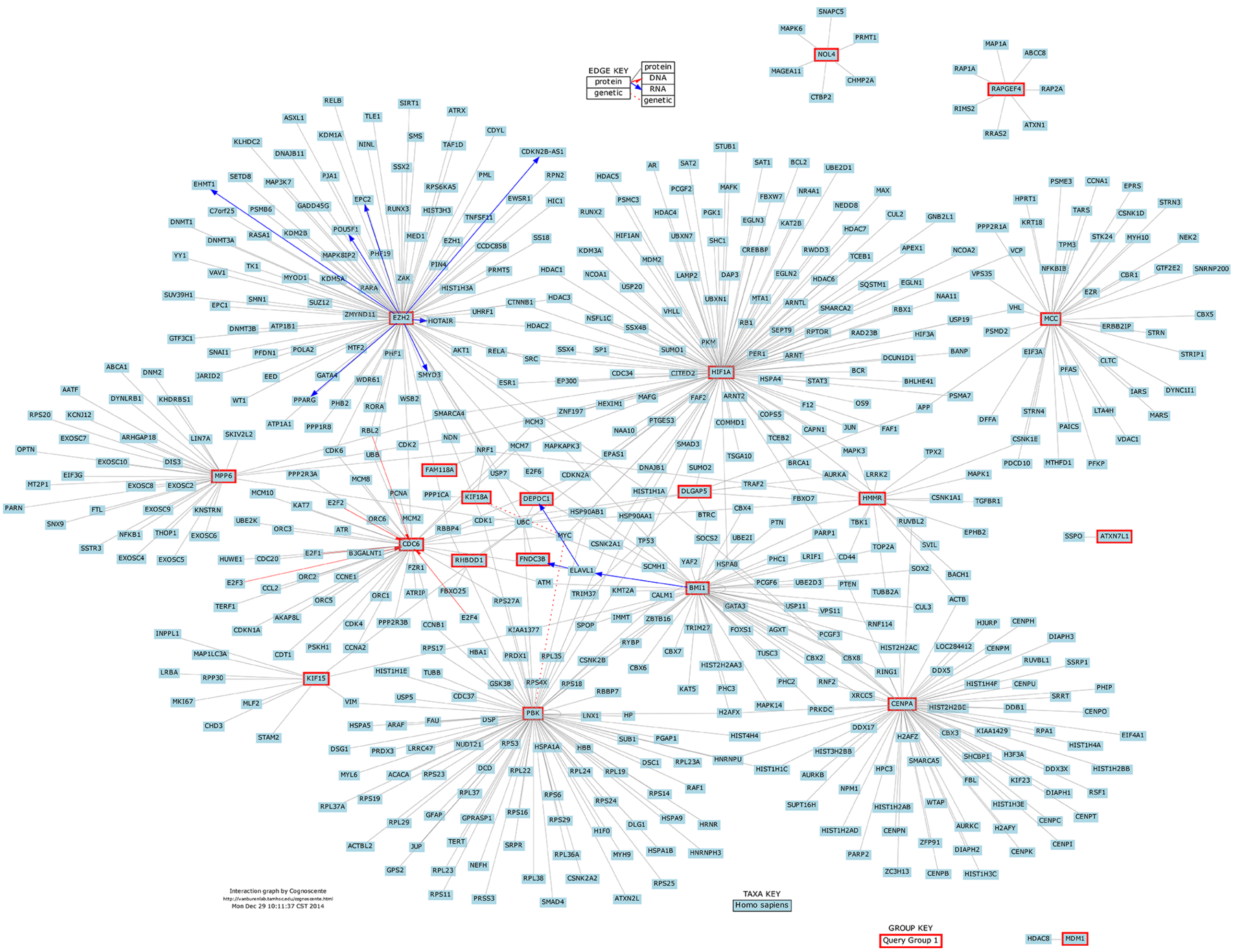

Supplementary Figure S8: Complete protein-protein interaction network (COGNOSCENTE) containing 20 selected genes, BMI1 and HIF1A.

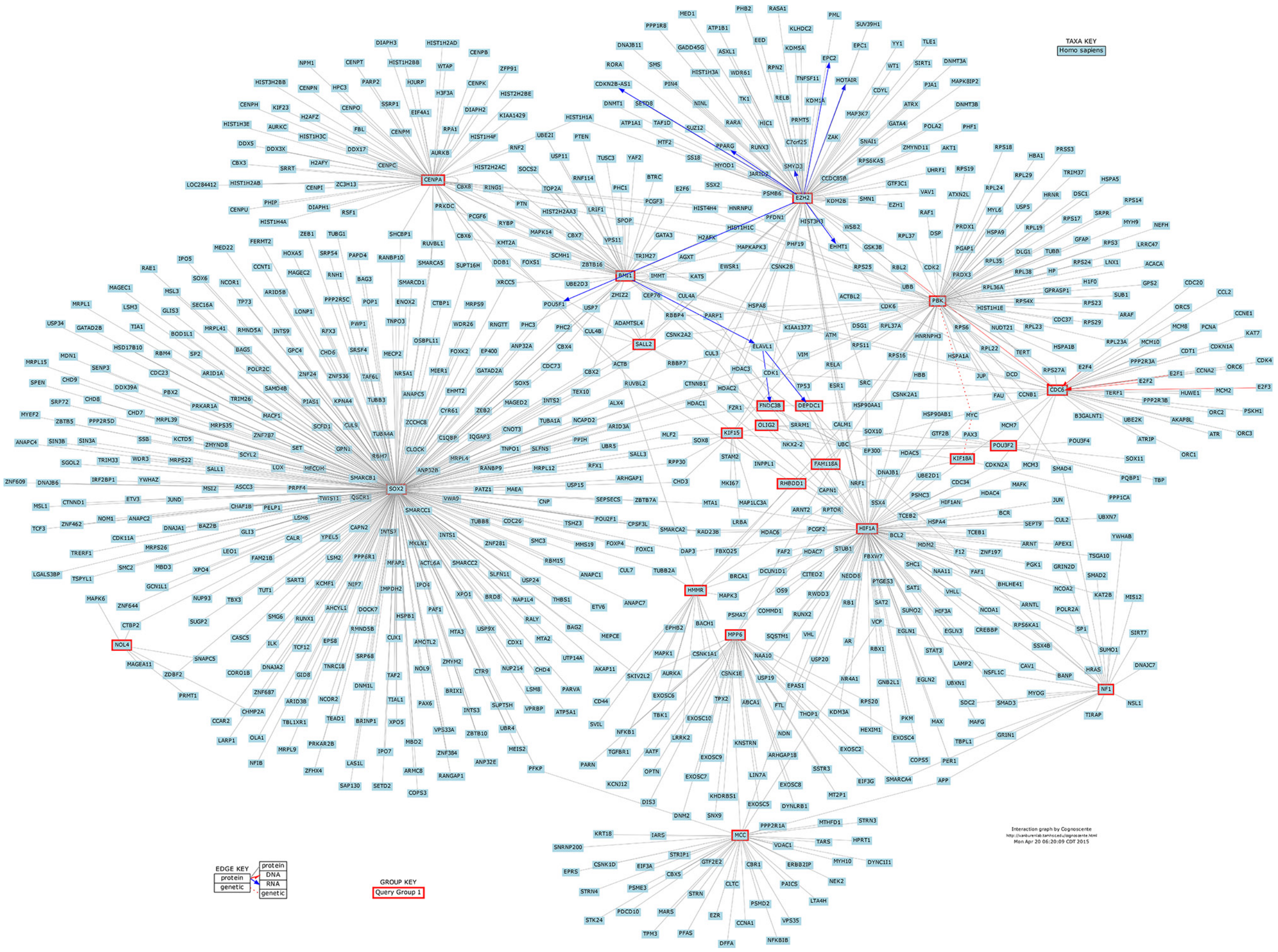

Supplementary Figure S9: Complete protein-protein interaction network (COGNOSCENTE) containing the selected 20 genes, BMI1 and HIF1A in addition to SALL2, POU3F2, OLIG2 and SOX2 depicted in Suva *et al*., 2014.

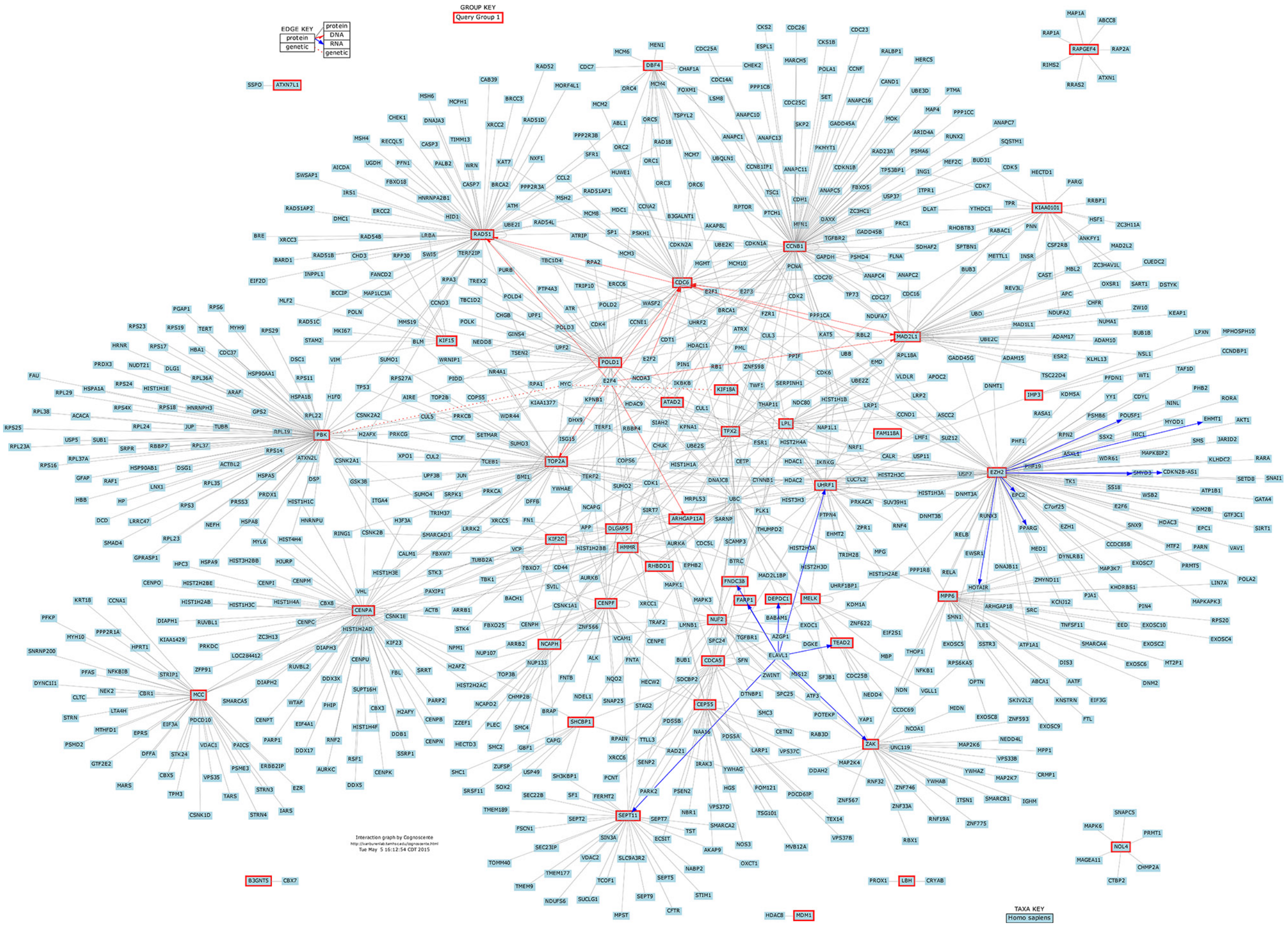

Supplementary Figure S10: Complete protein-protein interaction network (COGNOSCENTE) containing the selected 20 genes in addition to the 30-gene set from Sandberg *et al*., 2013.

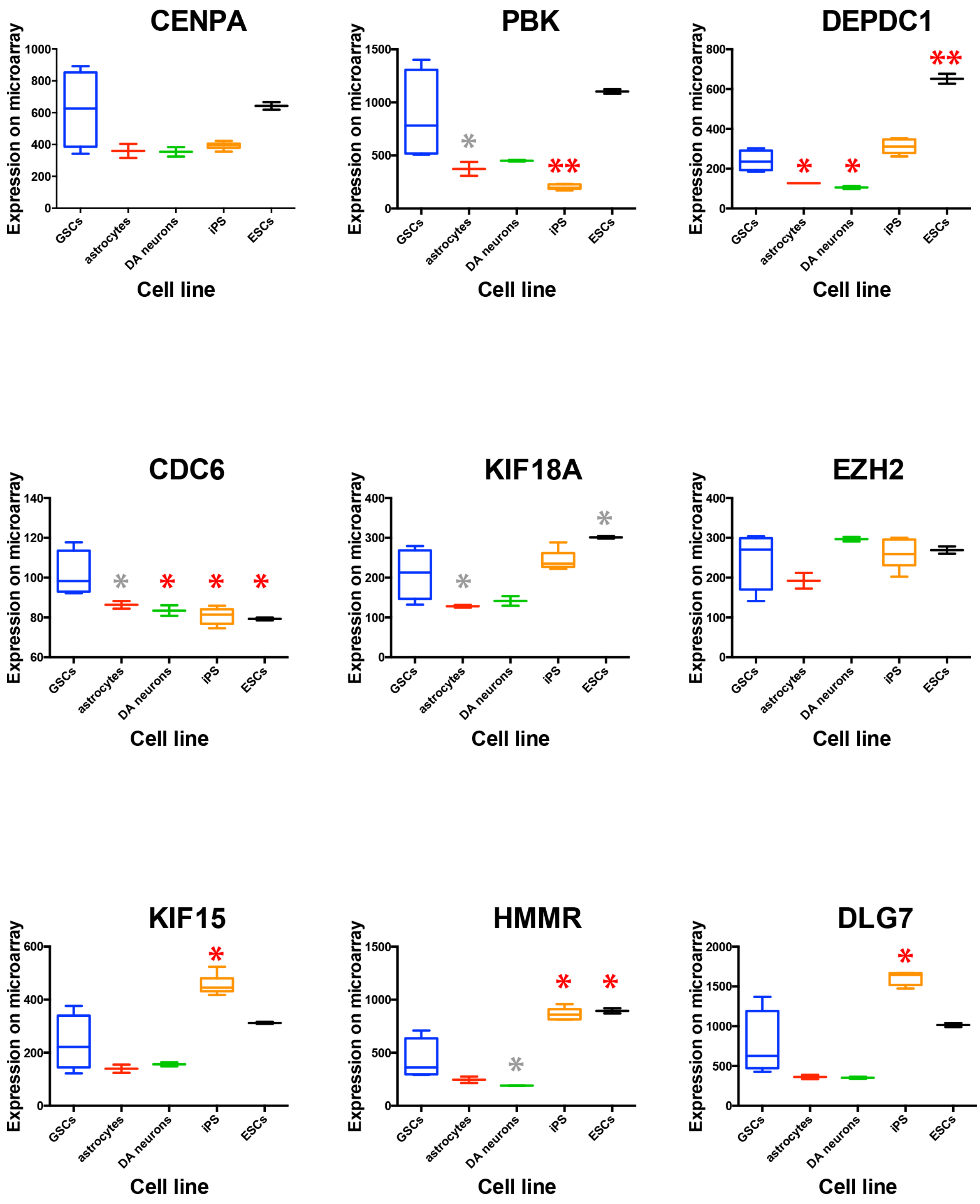

Supplementary Figure S11: The expression of *PBK, CENPA, KIF15, DEPDC1, CDC6, DLGAP5, KIF18A, EZH2 and HMMR* in GCS, astrocytes, DA-neurons, iPS-derived neurons, iPS cells and ESCs.

Supplementary Table S1: qPCR results with additional statistics

No.	GENE ID	Fold change	Std. Error	95% C.I.	P(H1)*	Result
1	CENPA	39,22	19.044 – 85.079	9.662 – 159.144	0.000	UP
2	DLG7	24,00	7.584 – 67.350	2.214 – 275.468	0.000	UP
3	PBK	38,06	15.996 – 93.012	7.612 – 231.414	0.000	UP
4	FILIP1L	4,52	0.560 – 77.704	0.188 – 184.341	0.095	NS
5	CDC6	9,19	3.098 – 21.116	1.411 – 31.312	0.000	UP
6	NOL4	30,53	7.156 – 160.174	0.182 – 518.565	0.000	UP
7	MCC	8,11	2.585 – 25.192	0.833 – 76.170	0.000	UP
8	KIF15	62,70	29.734 – 151.228	11.345 – 291.416	0.000	UP
9	MPP6	9,80	1.570 – 112.111	0.425 – 260.206	0.000	UP
10	KIF18A	10,99	4.482 – 31.906	0.140 – 48.410	0.000	UP
11	EZH2	35,31	12.521 – 82.809	4.725 – 218.897	0.000	UP
12	DEPDC1	15,68	4.755 – 51.315	2.446 – 135.096	0.000	UP
13	RAPGEF4	33,43	8.266 – 167.624	3.331 – 354.803	0.000	UP
14	HMMR	25,00	7.617 – 83.968	1.737 – 144.538	0.000	UP
15	ATXN7L4 v1	3,15	0.957 – 10.741	0.249 – 27.833	0.009	UP
15	ATXN7L4 v2	2,64	0.551 – 10.209	0.161 – 21.005	0.038	UP
16	P2RY5	0,50	0.227 – 1.091	0.133 – 2.661	0.057	NS
17	FAM118A	0,27	0.125 – 0.620	0.042 – 1.190	0.000	DOWN
18	RHBDD1	1,16	0.557 – 2.258	0.274 – 8.804	0.658	NS
19	FNDC3B	0,84	0.415 – 1.583	0.273 – 10.004	0.636	NS
20	MDM1	3,44	2.188 – 8.054	0.086 – 12.222	0.008	UP

**P* values and other statistical parameters were calculated using REST software (Qiagen) UP = up-regulated, DOWN = down-regulated, NS = not significant

Supplementary Table S2: The expression of the selected 20 genes in LGG and GBM

	GENE NAME	AFFY ID	RATIO	p.res	q.res	SIGNIF.
1	CENPA	204962_s_at	2.08833532	1.87E-007	6.91E-007	*
2	CENPA	210821_x_at	1.250145746	8.52E-008	4.09E-007	*
3	DLG7	203764_at	2.383889946	1.22E-008	6.53E-008	*
4	PBK	219148_at	3.286363179	6.17E-007	1.98E-006	*
5	FILIP1L	204135_at	1.366115233	0.004906417	0.009420321	*
6	FILIP1L	1554965_at	0.99371735	0.402561272	0.417834836	
7	FILIP1L	1554966_a_at	1.417337855	0.004114661	0.008229322	*
8	CDC6	203967_at	1.693819192	3.72E-009	2.89E-008	*
9	CDC6	203968_s_at	1.553137939	6.76E-009	4.06E-008	*
10	NOL4	206045_s_at	1.197917433	0.409129943	0.417834836	
11	NOL4	238605_at	0.828119874	0.000454627	0.000991914	*
12	MCC	206132_at	0.940831712	0.091144497	0.124998168	

(*Continued*)

	GENE NAME	AFFY ID	RATIO	p.res	q.res	SIGNIF.
13	MCC	226225_at	1.101582216	0.158150324	0.209587882	
14	KIF15	219306_at	1.924835596	4.37E-005	0.000110502	*
15	MPP6	205429_s_at	1.110288989	0.262382751	0.307179806	
16	KIF18A	221258_s_at	1.447925802	1.03E-006	3.08E-006	*
17	EZH2	203358_s_at	2.145984201	0.000158408	0.00038018	*
18	DEPDC1	220295_x_at	1.323502064	0.000273474	0.000625084	*
19	DEPDC1	222958_s_at	1.980617048	1.44E-010	1.73E-009	*
20	DEPDC1	232278_s_at	1.651068946	3.05E-009	2.89E-008	*
21	DEPDC1	235545_at	1.597512352	2.11E-007	7.22E-007	*
22	RAPGEF4	205651_x_at	0.768399337	0.014715382	0.023544611	*
23	HMMR	207165_at	2.474030022	7.96E-011	1.27E-009	*
24	HMMR	209709_s_at	1.430226051	1.40E-007	5.91E-007	*
25	HMMR	1562677_at	1.03851008	0.01340315	0.022184524	*
26	ATXN7L1	214342_at	0.990538098	0.362778593	0.391205997	
27	ATXN7L1	214343_s_at	1.027490857	0.161557326	0.209587882	
28	ATXN7L1	227732_at	1.154533873	0.00681203	0.012110275	*
29	ATXN7L1	232265_at	0.942766965	0.023441776	0.036296944	*
30	LPAR6	218589_at	1.354217912	0.001770903	0.003695797	*
31	LPAR6	1557763_at	0.952811432	0.048598212	0.06860924	*
32	MDM1	213761_at	1.059679136	0.463922172	0.463922172	
33	MDM1	220397_at	1.152197465	0.366755622	0.391205997	
34	FAM118A	219629_at	1.03064306	0.348782738	0.38933887	
35	FAM118A	226475_at	0.960994983	0.316793593	0.36204982	
36	RHBDD1	226945_at	0.968601984	0.19458384	0.245790114	
37	RHBDD1	226948_at	0.964699113	0.224991836	0.269990203	
38	RHBDD1	227414_at	0.922202035	0.043191176	0.064786764	*
39	RHBDD1	233164_x_at	0.924783164	0.006453943	0.011914972	*
40	RHBDD1	236690_at	0.971598188	0.208926689	0.257140541	
41	FNDC3B	218618_s_at	1.973386055	2.68E-006	7.57E-006	*
42	FNDC3B	222692_s_at	2.056943125	3.56E-011	1.27E-009	*
43	FNDC3B	222693_at	2.095871384	7.65E-011	1.27E-009	*
44	FNDC3B	225032_at	1.791224516	1.48E-007	5.91E-007	*
45	FNDC3B	229865_at	1.174187046	3.76E-005	0.000100243	*
46	FNDC3B	242029_at	1.389844657	4.21E-009	2.89E-008	*
47	FNDC3B	243830_at	0.943838151	0.047693557	0.06860924	*
48	FNDC3B	1569490_at	1.069213619	0.010292814	0.017644825	*

Expression of the 20 selected genes in GBMs and low-grade gliomas (LGG). Bioinformatic analysis of REMBRANDT database was done using 48 probes matching to the 20 genes. The up-regulation in GBM tissues is indicated as a ratio. Results were considered significant (asterisks) when *P*-values were <0.05.

Supplementary Table S3: Patient information (age, diagnosis and survival)

Tumor	Annotation	Sex	Age	Pathology	Post-surgical survival in months	Tumor localization	Comments
T0959	T59	M	48	GBM primary	10,5	Right temporal lobe	Tissue harvested from 1st surgery. Standard postop. chemo+radio
T0965	T65	M	61	GBM primary	15,2	Right parietal lobe	Tissue harvested from 1st surgery. Standard postop. chemo+radio
T0996	T96	F	62	GBM primary	12,2	Left parietooccipital lobe	Tissue harvested from 1st surgery. Standard postop. chemo+radio
T09111	T11	F	81	GBM secondary	1,5	Left temporal	Observed low-grade components and fields w/neovasc+necrosis, not given postopr. treatment due to comorbidity
T1008	T08	F	49	GBM primary	15,2	Right frontal lobe	1st stereotactic biopsy, then subtotal resection. Tissue from resection. Standard postop. chemo+radio
CAST #3	TC3	M	55	GBM primary	13,2	Right frontal lobe	Tissue harvested from 1st surgery. Vaccine pat. Standard postop. chemo+radio
CAST #4	TC4	M	64	GBM primary	10,4	Left parietal lobe	Tissue harvested from 1st surgery. Vaccine pat. Standard postop. chemo+radio. Multiple surgeries for abcesses
H0995		F	19	epilepsy	NA	NA	
H0991		F	43	epilepsy	NA	NA	
H1322		F	39	epilepsy	NA	NA	
H0980		F	53	epilepsy	NA	NA	
H1004		M	38	epilepsy	NA	NA	

Supplementary Table S4: List of oligonucleotides

NAME OF OLIGONUCLEOTIDE	SEQUENCE 5′-3
>Oli_1_DIR_CENPA_set1_1174	GGATGTGTAGCTTTTCAGAAACTTAAT
>Oli_2_REV_CENPA_set1_1390	GAAACTACAAAAATACCCATAAAAGCA
>Oli_3_DIR_CENPA_set2_476	AGAAGCATTTCTAGTTCATCTCTTTGA
>Oli_4_REV_CENPA_set2_756	ATTTAAAGCAACACACACATACTGTTC

(*Continued*)

NAME OF OLIGONUCLEOTIDE	SEQUENCE 5′-3
>Oli_5_DIR_CENPA_set3_285	GCTTCCTCCCATCAACACAGTC
>Oli_6_REV_CENPA_set3_489	CTAGAAATGCTTCTGCTGCCTCT
>Oli_7_DIR_PBK_set1_1515	TGGATCTACTGACATTAGCACTTTGTA
>Oli_8_REV_ PBK _set1_1844	CCAAAGTGTCCTTTATTCTTTATCATC
>Oli_9_DIR_ PBK _set2_904	TTACTTTGTGGGAAATGATGACTTTAT
>Oli_10_REV_ PBK _set2_1107	CATTAGTGCATACAGAGAAGAGTTCAA
>Oli_11_DIR_RAPGEF4_set1_3695	TAGAAAATAACATTTCAGAAGAGCACA
>Oli_12_REV_RAPGEF4_set1_4024	TGTAGTCTAATTGTGAAAACAGGAAAA
>Oli_13_DIR_RAPGEF4_set2_1947	GATGAAGTTCTGTTTAAGGTCTATTGC
>Oli_14_REV_RAPGEF4_set2_2296	TCATAAATTGTCATCTGGTATGCTAAA
>Oli_15_DIR_RAPGEF4_set3_368	AGAAGGACGTATGCTCTACAAGAAATA
>Oli_16_REV_RAPGEF4_set3_622	TTTCTATCTCTTATCATGTGAGGTGCT
>Oli_17_DIR_DLG7_set1_2525	AAAATACAGCTTCACAAAATAGCATCT
>Oli_18_REV_ DLG7_set1_2846	ACAAATACATTTTCTCCAAAATTTCAA
>Oli_19_DIR_ DLG7_set2_2144	TCAACAAAGCTGTATCTCAGAGTAGAA
>Oli_20_REV_ DLG7_set2_2368	AATCCACCTTCAAGTCTGTCTTATTTA
>Oli_21_DIR_ DLG7_set3_1105	AATACTGCAAAAATAAAAGGGAAGAAT
>Oli_22_REV_ DLG7_set3_1445	ATTGGAAGGCCATTTAAATTTTTAGTA
>Oli_23_DIR_CDC6_set1_2752	TGGGGTCATAAGGAGACTATAACTCTA
>Oli_24_REV_CDC6_set1_3016	TGATTAACCAACCATATGGACTAATTT
>Oli_25_DIR_CDC6_set2_1936	AGAGAGCTACAGTCTTCATTTTAGTGC
>Oli_26_REV_CDC6_set2_2207	CGAAGAGGTAAATATACACACATTCCT
>Oli_27_DIR_CDC6_set3_238	CTACAATCAGTTTTCCAAAAAGGAAG
>Oli_28_REV_CDC6_set3_574	GAAAGTATTTTGTTTTGGTGAACTTTG
>Oli_29_DIR_NOL4_set1_3510	TGAGGCATCAGTGATATTTCTTATCTA
>Oli_30_REV_NOL4_set1_3728	AGATTTTTCACATTATATTCACCAACA
>Oli_31_DIR_NOL4_set2_2247	TTAAGAGAAATACTTCCATTATGCCAC
>Oli_32_REV_NOL4_set2_2505	AGTAGCAAATTCCACTAATATTTCAGC
>Oli_33_DIR_FILIP1L_set1_3632	CTGAGGATAATAAAATCCACATTCACT
>Oli_34_REV_FILIP1L_set1_3947	TTTTTAATAAATGCTTAACCCAGTTCA
>Oli_35_DIR_FILIP1L_set2_2705	AAAAACTAAATCAACAAGAAAACAGGA
>Oli_36_REV_FILIP1L_set2_2958	CTCACTCTCCTCATATAACTGACCATT
>Oli_37_DIR_FILIP1L_set3_1713	TGAAAAACTCAGTAAAAGAATTATGGC
>Oli_38_REV_FILIP1L_set3_1921	TTAGAGTGAATTCTGTCTTTTCTAGCC
>Oli_39_DIR_FILIP1L_set4_897	ACAAAGTTGTGGAAAAACATAAAGAAT
>Oli_40_REV_FILIP1L_set4_1100	GACTTGATTTCTTGATCAATTAGCTTC
>Oli_41_DIR_MPP6_set1_1784	ATTCAGAGAGCATACAACCACTATTTT
>Oli_42_REV_ MPP6_set1_2109	TATTAGTACACCTTCCAACCACATTTT

NAME OF OLIGONUCLEOTIDE	SEQUENCE 5′-3
>Oli_43_DIR_ MPP6_set2_930	ATACCATTACTCCTCAACAGGTATTTG
>Oli_44_REV_ MPP6_set2_1168	GTCTCTTCTAACAAATGCCTTTCTCTT
>Oli_45_DIR_MCC_set1_8226	TAATGCAAAGATAATAAAACATGTCCA
>Oli_46_REV_MCC_set1_8555	TATTTTACAGCAGCTAAAACTAAAGGC
>Oli_47_DIR_MCC_set2_6972	AATAGCTCTCAAGAGTTTCGATTATCA
>Oli_48_REV_MCC_set2_7213	GAAGTCTCTTACTATTTTGGCTCTCAA
>Oli_49_DIR_MCC_set3_5802	TTACTCATGCTACCTGACCTTAGTTTT
>Oli_50_REV_MCC_set3_6146	TTAGAATCAAGATCATTGTTTACCCTC
>Oli_55_DIR_MCC_set6_2029 EXO2	TGATTTTTCTTATCTCTACTGGCTTTC
>Oli_56_REV_MCC_set6_2363 EXO2	TTAGTAGCAGAGTCAAATTCGGTCTAT
>Oli_57_DIR_MCC_set7_573	GATACATCAGCAGAAATGACTTGCTA
>Oli_58_REV_MCC_set7_781	CTTCTTTGTACAGGAGTTGTCTGACTT
>Oli_59_DIR_KIF15_set1_4563	TTTGTCTTTGTAAAAATAAAAGCCTGT
>Oli_60_REV_KIF15_set1_4817	CTTTATTTACAGTCATGGAAAATGCTC
>Oli_61_DIR_KIF15_set2_3504	ATGAATATAACTTCAAAATGAGGCAAC
>Oli_62_REV_KIF15_set2_3706	GACTTCTCTGTCTTCATTTAGCTTTGT
>Oli_63_DIR_KIF18A_set1_2268	TTAGCAAAGAACTTCAGCCTATTGTAT
>Oli_64_REV_KIF18A_set1_2575	TTGTAAAATGTCTTTGTTATCATTTGG
>Oli_65_DIR_EZH2_set1_2129	ACTGCTATGCAAAAGTTATGATGGTTA
>Oli_66_REV_EZH2_set1_2462	GTGAGAAGGCAATAAAAAGTTGATTT
>Oli_67_DIR_DEPDC1_set1_4188	TAATGACCCACTCTTCTTATGTTATCC
>Oli_68_REV_DEPDC1_set1_4454	AGAGATCTTAAACTCAATACAAGGCAA
>Oli_69_DIR_HMMR_set1_2823	CAGTTCAAATAAGAAATAAGGACAAGC
>Oli_70_REV_HMMR_set1_3101	ATTACACAAATGTTAATCAAATGCTGA
>Oli_71_DIR_P2RY5_set1_1802	GTAAGATATTTGACAATGAATCTGCTG
>Oli_72_REV_P2RY5_set1_2150	AAAGGAATTCAAAGACATTACAGATTG
>Oli_73_DIR_ATXN7L1_set1_5436	TTGTATGCCTTCTTTTGTATCAATGTA
>Oli_74_REV_ATXN7L1_set1_5685	TGCAACAACAGTTATTTAATATACCCC
>Oli_75_DIR_ATXN7L1V2_set2_1327	CAGTGAGGCTAGTTGAGAAATTATACC
>Oli_76_REV_ATXN7L1V2_set2_1650	ATCCAATAGATGTTTAATGACCCAGT
>Oli_77_DIR_FAM118A_set1_2766	TGTTATTGTCTCTTAAAATTCTCTTCCA
>Oli_78_REV_FAM118A_set1_3106	TTATCTGTACCTATGGGAGTCATCAAT
>Oli_79_DIR_RPL30_228	AGCTTTGAGGAAATCTGAAATAGAGTA
>Oli_80_REV_RPL30_441	GTGAAATTTTGTAGGTGAAAAGGTTTA
>Oli_81_DIR_RPL27_226	GTATAACTACAATCACCTAATGCCCAC
>Oli_82_REV_RPL27_477	TTTAATGATCAAAACAAAGCATCTAAA
>Oli_93_DIR_GAPDH_set1_1040	GTATGACAACGAATTTGGCTACAG
>Oli_94_REV_GAPDH_set1_1296	GTACTTTATTGATGGTACATGACAAGG

(*Continued*)

NAME OF OLIGONUCLEOTIDE	SEQUENCE 5′-3
>Oli_117_DIR_GFAP_V2_set1_1523	AGTAACTGTACATTAAACTGGCAGAGC
>Oli_118_REV_GFAP_V2_set1_1817	TAACATTAAGAGCAGGGAACATAAAAC
>Oli_123_DIR_GFAP_V1_set2_2101	GATGACTCAAGTGTCTCAGTCCAC
>Oli_124_REV_GFAP_V1_set2_2434	GAGTCACTTCCTTAATTCCCACAAT
>Oli_129_DIR_NES_set1_5247	CTAAGTCAGCTGAATCCCGATAGTA
>Oli_130_REV_NES_set1_5544	CATTACTTTATTCAGGCAGGGACTA
>Oli_135_DIR_TUB_set1_1368	AGATGTACGAAGACGACGAGGAG
>Oli_136_REV_TUB_set1_1712	TTAAGGGTATCTGACAGCAATAGATTT
>Oli_161_DIR_NOL4_set3_1460	TTAATATGTTTGTCAGGCTGTTTGTAG
>Oli_162_REV_NOL4_set3_1659	AGACATCTCAAAACCACTTCTTTTCAT
>Oli_368_DIR_RHBDD1_set1_4646	TTCTACATAACTCCAGCTTAGTCTTCC
>Oli_369_REV_RHBDD1_set1_4982	ACTGTGTAGATGTGGTTGTGTCTGTTA
>Oli_370_DIR_FNDC3B_set1_6496	TGACTCTTCTCTGTATGTCAAACTCAT
>Oli_371_REV_FNDC3B_set1_6840	CATGATTTTCTTTTTCTCCTAGAGTTG
>Oli_271_DIR_MDM1V1_set1_2472	ACATCTATCCCAGAGTACAAAGACCTA
>Oli_272_REV_MDM1V1_set1_2780	AGCTGGTGTTTTCAAAGTACAATTATC

The oligonucleotide-sets for the detection of the 20 candidate genes were designed to cover most of the transcribed sequences. This was done by designing one to seven sets of oligonucleotides per gene, starting from the 3'UTR end and progressing towards the 5' end of the gene with 1–2 kb distance. Remark: We designed our own oligonucleotides for housekeeping genes RPL27 and RPL30. Use of RPL27 and RPL30 as controls for normalizing qPCR results was suggested by de Jonge et al. (de Jonge HJ, Fehrmann RS, de Bont ES, Hofstra RM, Gerbens F, Kamps WA, de Vries EG, van der Zee AG, te Meerman GJ, ter Elst A: Evidence based selection of housekeeping genes. PloS One 2007; 2:e898.)

Supplementary Table S5: List of primary and secondary antibodies used for western blot

PRIMARY ANTIBODIES WESTERN					
	NAME OF THE ANTIBODY	PRODUCT NUMBER	COMPANY	ANIMAL	DILUTION
1	ACTIN	#4967	Cell Signaling Technology	rabbit	1:1000
2	ATXN7L1	SAB2100189	Sigma-Aldrich	rabbit	1:1000
3	CENPA	#2186	Cell Signaling Technology	rabbit	1:1000
4	CDC6	C42F7	Cell Signaling Technology	rabbit	1:1000
5	FAM118A	SAB1102078	Sigma-Aldrich	rabbit	1:250
6	DEPDC1	WH0055635M5	Sigma-Aldrich	mouse	1:500
7	RHBDD1	SAB3500406	Sigma-Aldrich	rabbit	1:500
8	DLG7	HPA005546	Sigma-Aldrich	rabbit	1:75
9	FILIP1L	HPA043133	Sigma-Aldrich	rabbit	1:200

(*Continued*)

PRIMARY ANTIBODIES WESTERN					
	NAME OF THE ANTIBODY	PRODUCT NUMBER	COMPANY	ANIMAL	DILUTION
10	EZH2	#5246	Cell Signaling Technology	rabbit	1:1000
11	FNDC3B	HPA007859	Sigma-Aldrich	rabbit	1:250
12	HMMR	EPR4055/ ab108339	Abcam	rabbit	1:1000
13	KIF15	WH0056992M1	Sigma-Aldrich	mouse	1:200
14	KIF18A	ab72417	Abcam	rabbit	1:2000
15	MCC	WH0004163M1	Sigma-Aldrich	mouse	1:500
16	MDM1	HPA041594	Sigma-Aldrich	rabbit	1:1000
17	MPP6	HPA019085	Sigma-Aldrich	rabbit	1:1000
18	NOL4	WH0008715M1	Sigma-Aldrich	mouse	1:500
19	PBK	#4942	Cell Signaling Technology	rabbit	1:1000
20	P2RY5	sc-20126	Santa Cruz Biotechnology	goat	1:500
21	RAPGEF4	WH0011069M1	Sigma-Aldrich	mouse	1:1000
22	EGF Receptor	D38B1	Cell Signaling Technology	rabbit	1:1000
23	SHH	MAB464	RD Systems	rat	1:500
24	GLI1	AF3455	RD Systems	goat	1:1000
25	GFAP (GA5)	#3670	Cell Signaling Technology	mouse	1:1000
26	TUBB3	T8660	Sigma-Aldrich	mouse	1:500
27	NESTIN	MAB5326	Millipore	mouse	1:1000
28	IGFBP5	NBP1-50718	Novus	rabbit	1:1000
29	OCT4	AF1759	RD Systems	goat	1:1000
30	mTOR	#2983	Cell Signaling Technology	rabbit	1:1000
31	pSTAT3(pSer727)	SAB300034	Sigma-Aldrich	rabbit	1:1000
32	DLL3	#2483	Cell Signaling Technology	rabbit	1:1000
33	PDGFB	SAB2101755-50UG	Sigma-Aldrich	rabbit	1:1000
34	CTNNB	610153	BD transduction Laboratories	mouse	1:500
35	ABC Active®-Catenin	#05-665	Millipore	mouse	1:500
36	PDGFR	#5241	Cell Signaling Technology	rabbit	1:1000

(*Continued*)

	NAME OF THE ANTIBODY	PRODUCT NUMBER	COMPANY	ANIMAL	DILUTION
**PRIMARY ANTIBODIES WESTERN**					
37	SOX2	MAB2018	RD Systems	mouse	1:500
38	NF1	MAB5438	Millipore	mouse	1:500
**SECONDARY ANTIBODIES WESTERN**					
1	ECL Anti-rabbit IgG-HRP	NA934	Amersham		1:10000
2	ECL Anti-mouse IgG-HRP	NA931	Amersham		1:10000
	Anti-mouse IgG-HPR	#7076	Cell Signaling Technology		1:10000
3	Anti-goat IgG-HRP	sc-2020	Santa Cruz Biotechnology		1:10000
4	Anti-rat IgG	NA935	Amersham		1:10000

Supplementary Table S6: List of primary and secondary antibodies used for immunolabeling

	NAME	PRODUCT NUMBER	COMPANY	ANIMAL	DILUTION
**PRIMARY ANTIBODIES-IMMUNOLABELING**					
1	CENPA	ab20665	Abcam	rabbit	1:50
2	DLG7	HPA005546	Sigma-Aldrich	rabbit	1:75
3	MDM1	H00056890	Abnova	mouse	1:500
4	MPP6	ab85718	Abcam	goat	1:100
5	HMMR	ab67003	Abcam	mouse	1:50
6	PBK	#612170	BD Transduction Laboratories	mouse	1:100
7	KIF15	WH0056992M1	Sigma-Aldrich	mouse	1:200
8	KIF18A	ab72417	Abcam	rabbit	1:500
9	CDC6	ab64074	Abcam	mouse	1:75
10	EZH2	AF4767	RD Systems	goat	1:100
11	NES	#ab6320	Abcam	mouse	1:400
12	TUBB3	T8660	Sigma-Aldrich	mouse	1:400
13	GFAP	#3670	Cell Signaling Technology	mouse	1:300
**SECONDARY ANTIBODIES-IMMUNOLABELING**					
1	Anti-rabbit Alexa 488	A11008	Molecular Probes Invitrogen	goat	1:500
2	Anti-mouse Alexa 594	A11005	Molecular Probes Invitrogen	goat	1:500
3	Anti-goat Alexa 594	A11058	Molecular Probes Invitrogen	donkey	1:500
4	Anti-rabbit Alexa 594	A11012	Molecular Probes Invitrogen	goat	1:500
5	Anti-mouse Alexa 647	A-21236	Molecular Probes Invitrogen	goat	1:500
6	Anti-goat Alexa 488	A11055	Molecular Probes Invitrogen	donkey	1:500
